# Supplementary material for: Cerebral Malaria Model Applying Human Brain Organoids
Source: Cells. 2023 Mar 23;12(7):984. doi: 10.3390/cells12070984 (PMC10093648; doi:10.3390/cells12070984)
Supplement: Supplementary file 1 [file cells-12-00984-s001.zip › cells-2141809-supplementary.pdf]

Supplementary Material

A

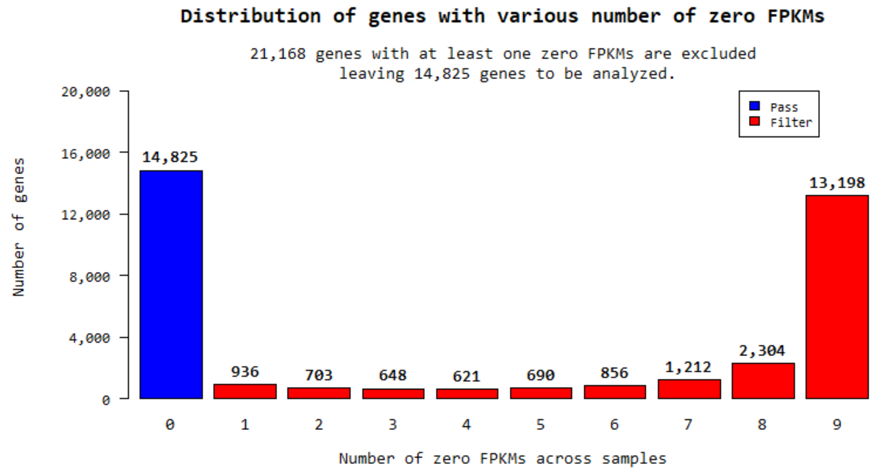

B

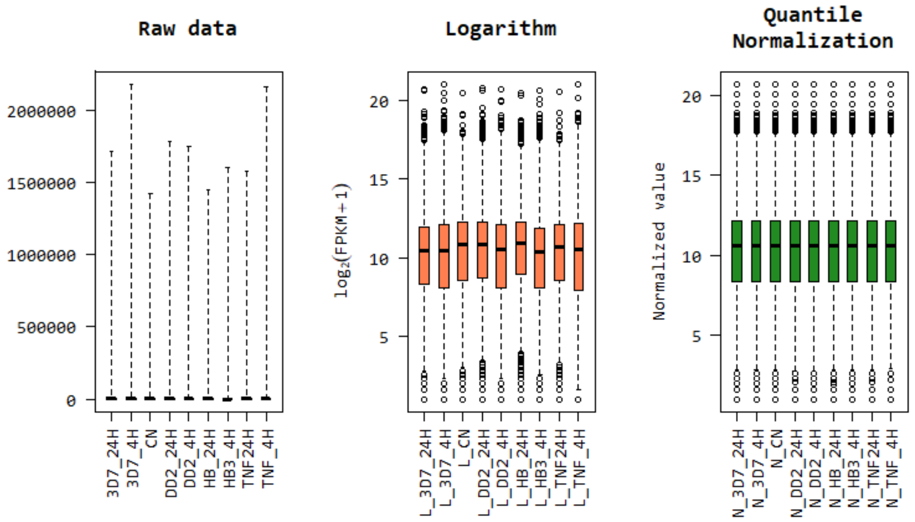

C

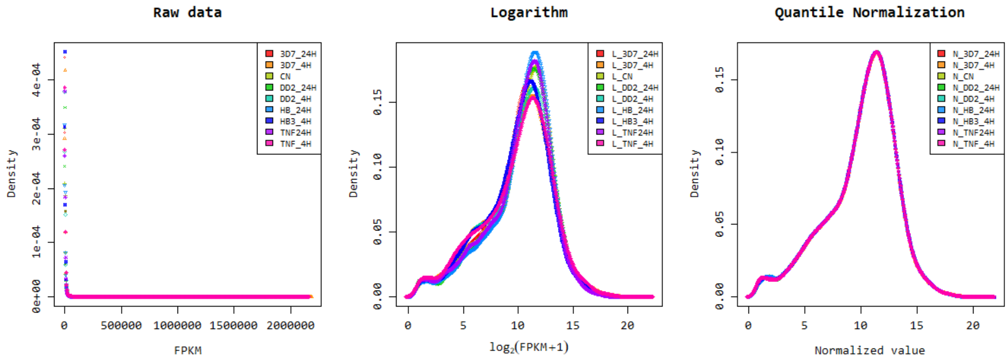

D

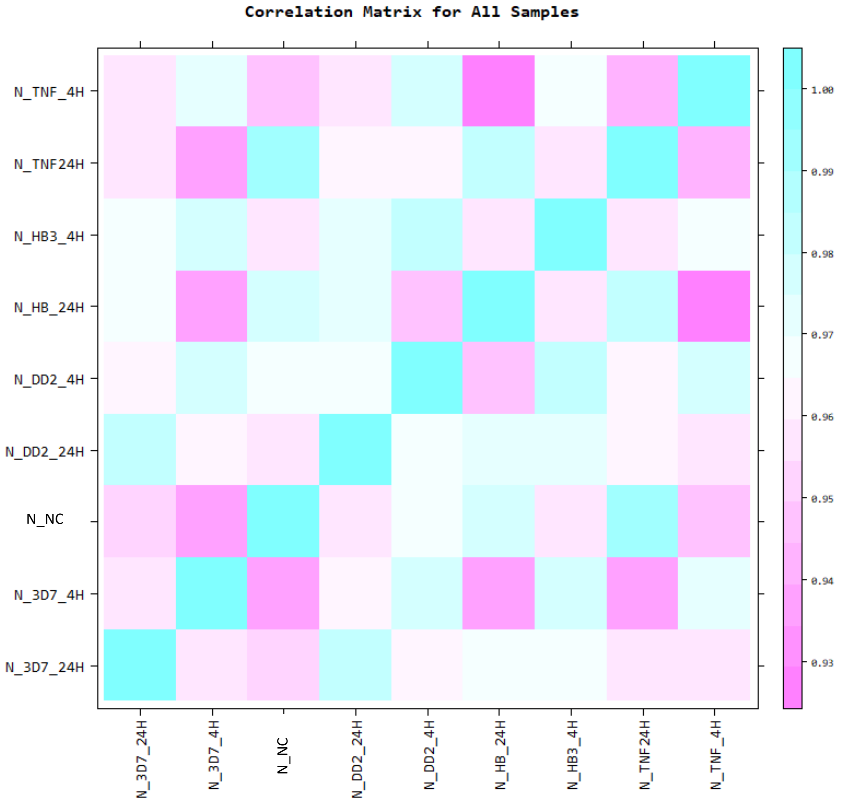
Correlation Matrix for All Samples
N_TNF_4H
N_TNF24H
N_HB3_4H
N_HB_24H
N_DD2_4H
N_DD2_24H
N_NC
N_3D7_4H
N_3D7_24H
N_3D7_24H
N_3D7_4H
N_NC
N_DD2_24H
N_DD2_4H
N_HB_24H
N_HB3_4H
N_TNF24H
N_TNF_4H
1.00
0.99
0.98
0.97
0.96
0.95
0.94
0.93

E

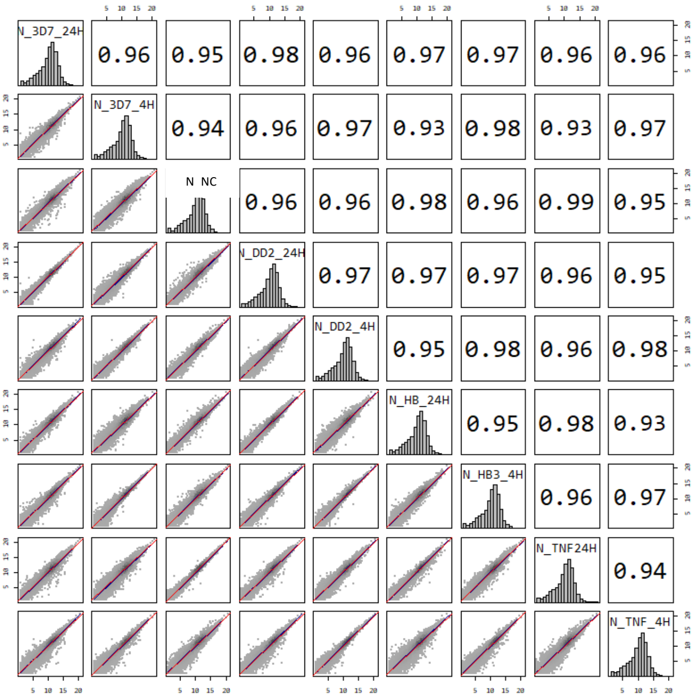
N_3D7_24H
0.96 0.95 0.98 0.96 0.97 0.97 0.96 0.96
N_3D7_4H
0.94 0.96 0.97 0.93 0.98 0.93 0.97
N_NC
0.96 0.96 0.98 0.96 0.99 0.95
N_DD2_24H
0.97 0.97 0.97 0.96 0.95
N_DD2_4H
0.95 0.98 0.96 0.98
N_HB_24H
0.95 0.98 0.93
N_HB3_4H
0.96 0.97
N_TNF24H
0.94
N_TNF_4H

F

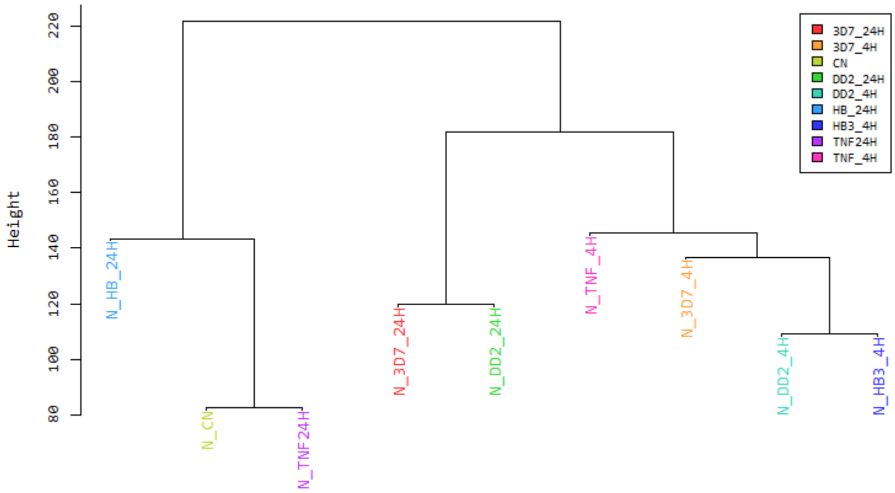

Figure S1: HBMEC transcriptome data. (A) The data quality is evaluated through QC for each gene (histogram): For 9 samples, if more than one FPKM value was 0, it was not included in the analysis. Therefore, from the total of 35,993 genes, 21,168 were excluded, and only 14,825 genes were used for statistical analysis. (B) Box plot: Below boxplots show the corresponding sample's expression distribution based on a percentile (median, 50 percentile, 75 percentile, maximum, and minimum) based on raw signal (FPKM), Log2 transformation, and Quantile Normalization. (C) Density plot: Below density plots show the corresponding sample's expression distribution before and after raw signal (FPKM+1), Log2 transformation, and Quantile Normalization. (D) Correlation matrix between Samples: The similarity between samples is obtained through Pearson's coefficient of the sample's normalized value. For range: -1⩽ r ⩽ 1, the closer the value is to 1, the more similar the samples were. The correlation matrix of all samples is as follows (X-axis as control and Y-axis as a test is the average normalized value of the group). (E) Reproducibility between samples, the closer the value is to 1, the more similar the samples are. (F) Hierarchical clustering (distance metric = Euclidean distance, linkage method = complete) analysis and graphically represents the similarity of expression patterns between all samples and genes. The samples in the analysis are HBMEC without stimulation, used as negative control (NC), HBMEC with

stimulation, working as a positive control, and TNF-α at 4 h (TNF_4h) and 24 h (TNF_24h). HBMEC stimulated with Dd2 (Dd2_4h Dd2_24h), HB3 (HB3_4h, Hb3_24h), and 3D7 (3D7_4h, 3D7_24h) strains of *P. falciparum* parasites at a time point of 4 h and 24 h, respectively.

A – Expression level of the genes between 3D7_4h_vs_NC

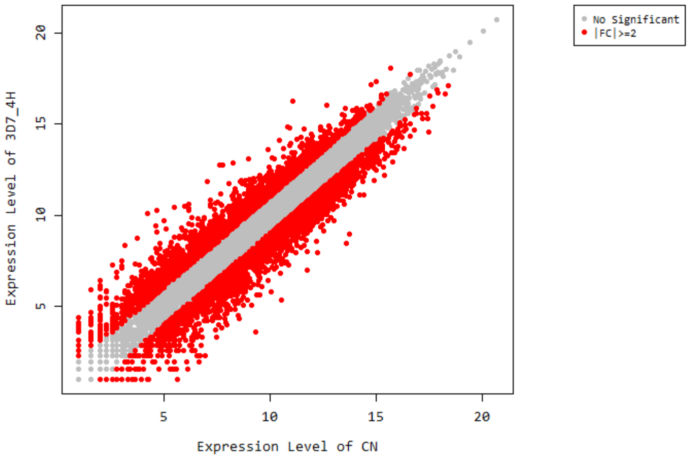

B – Expression level of the genes between 3D7_24h_vs_NC

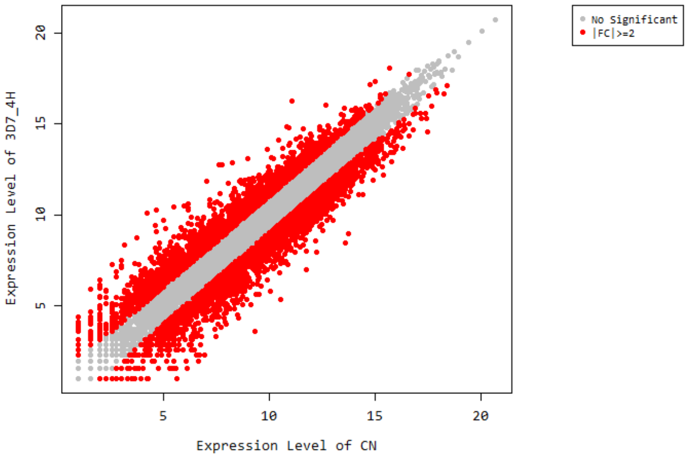

C – Expression level of the genes between Dd2_4h_vs_NC

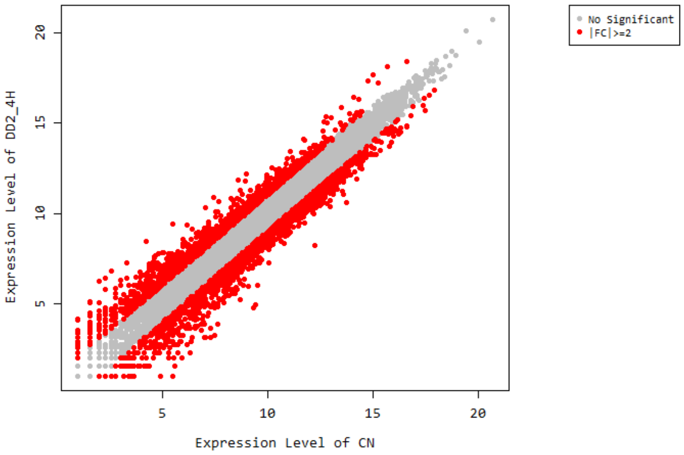

D – Expression level of the genes between Dd2_24h_vs_NC

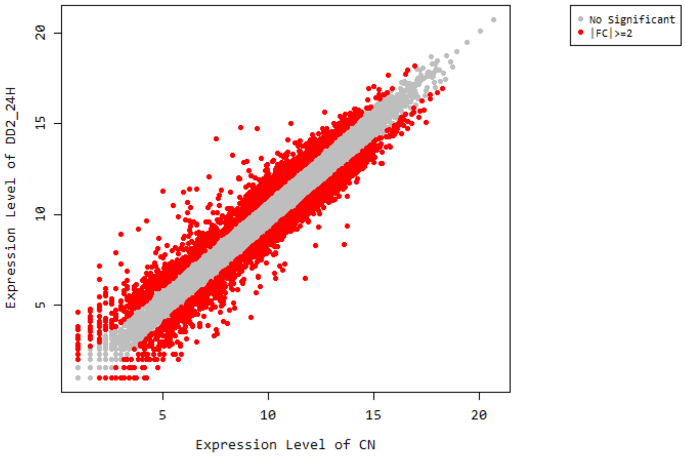

E – Expression level of the genes between HB3_4h_vs_NC

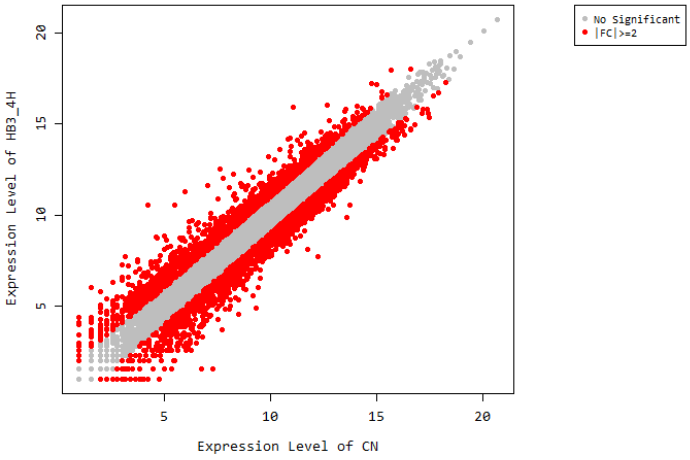

F – Expression level of the genes between HB3_24h_vs_NC

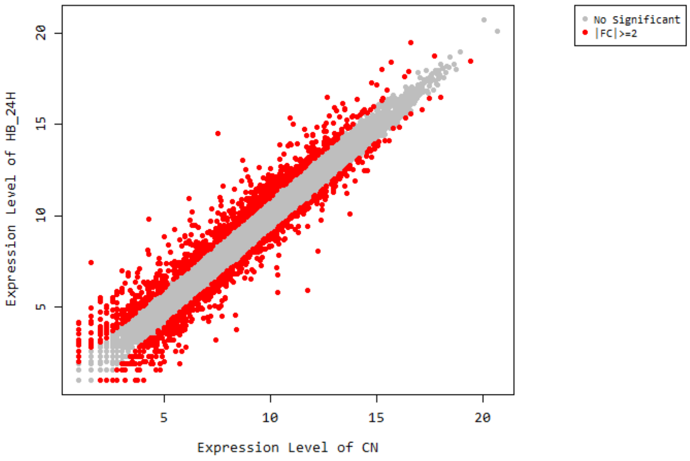

G – Expression level of the genes between TNF-α_4h_vs_NC

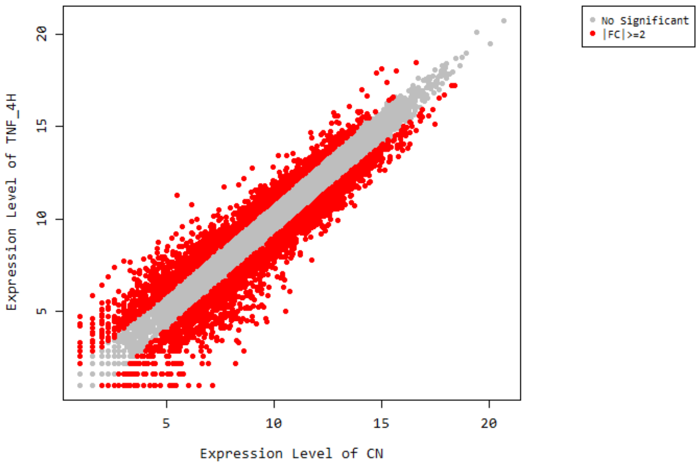

H - Expression level of the genes between TNF-α_24h_vs_NC

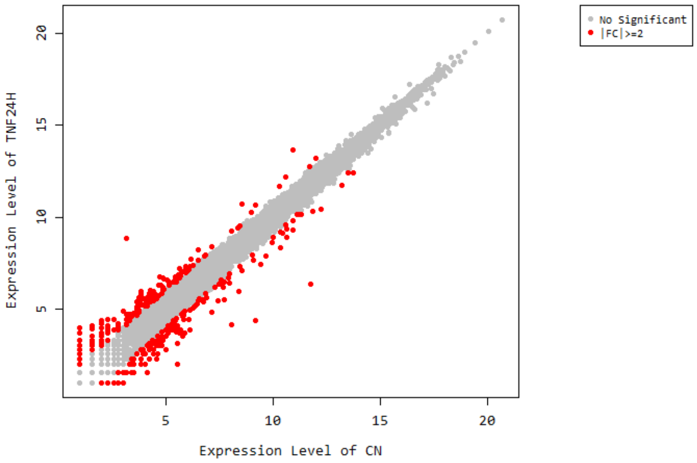

Figure S2: Scatter plot of expression level. Shows distribution of normalized expression levels values per group between comparison pairs as a scatter plot by 2-fold-change. The samples in the analysis are HBMEC without stimulation, used as negative control (NC), HBMEC with stimulation, working as a positive control, TNF

at 4 h (TNF_4h) and 24 h (TNF_24h). HBMEC stimulated with Dd2 (Dd2_4h Dd2_24h), HB3 (HB3_4h, Hb3_24h), and 3D7 (3D7_4h, 3D7_24h) strains of *P. falciparum* parasites at a time point of 4 h and 24 h, respectively. The scatter plot of the genes expression level between NC and (A) 3D7 4H, (B) 3D7 24H, (C) Dd2 4H, (D) Dd2 24H, (E) HB3 4H, (F) HB3 24H, (G) TNF 4H, (H) TNF 24H.

A - Volume Plot Between 3D7_4h_vs_NC

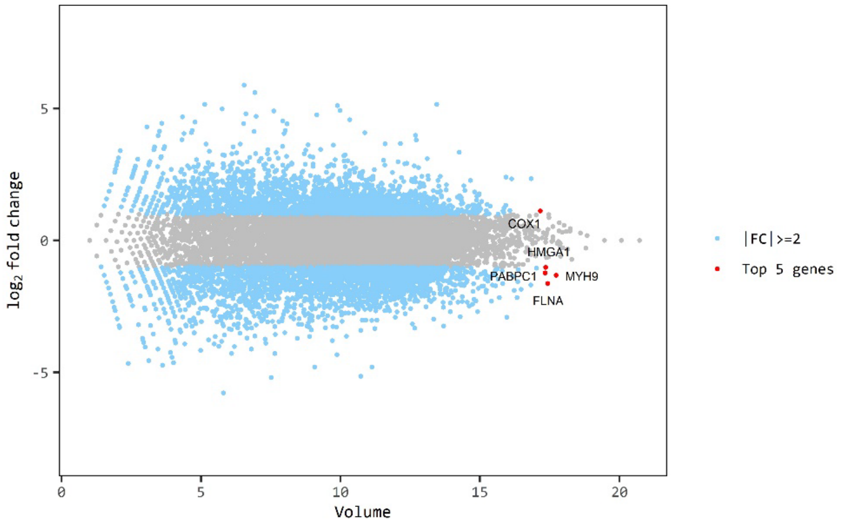

B - Volume Plot Between 3D7_24h_vs_NC

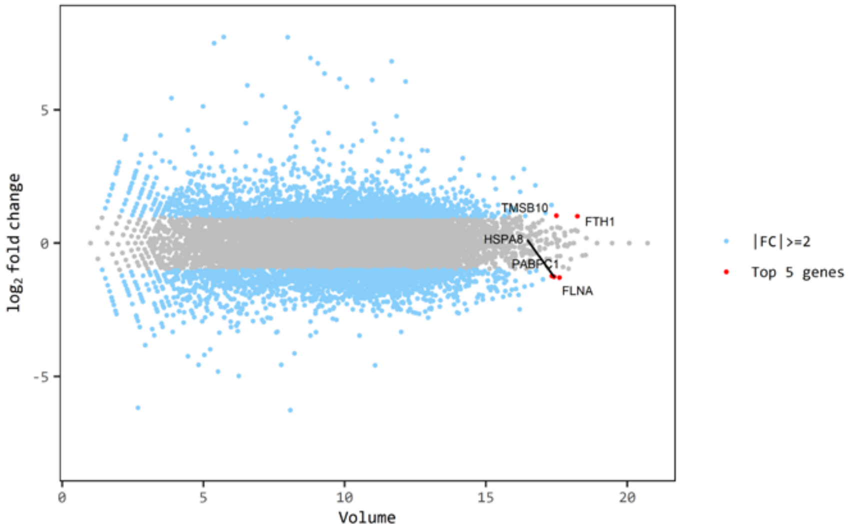

C - Volume Plot Between Dd2_4h_vs_NC

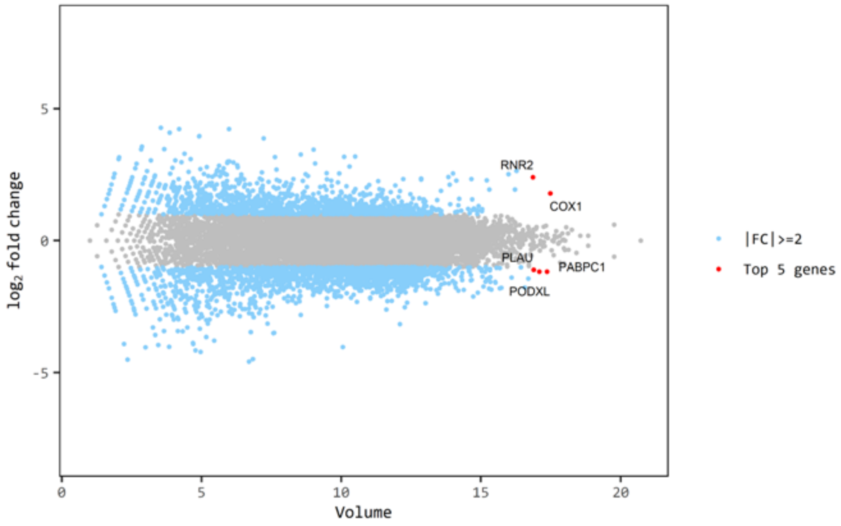

D - Volume Plot Between Dd2_24h_vs_NC

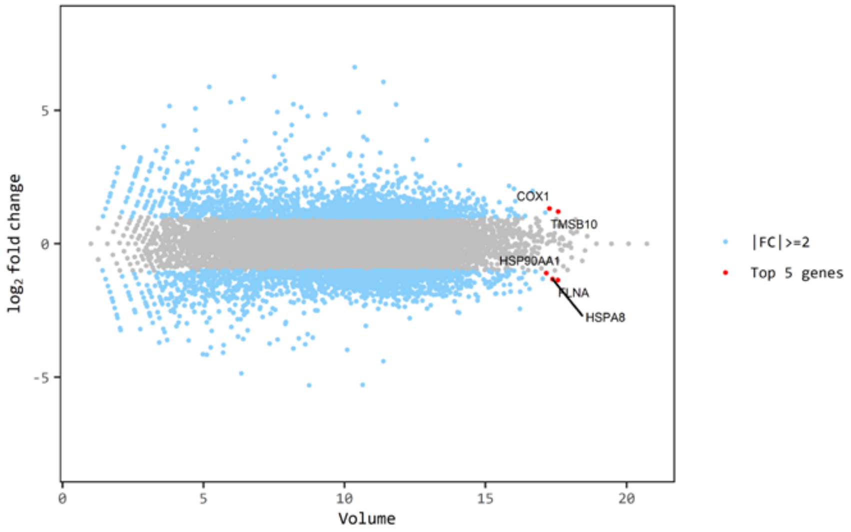

E - Volume Plot Between HB3_4h_vs_NC

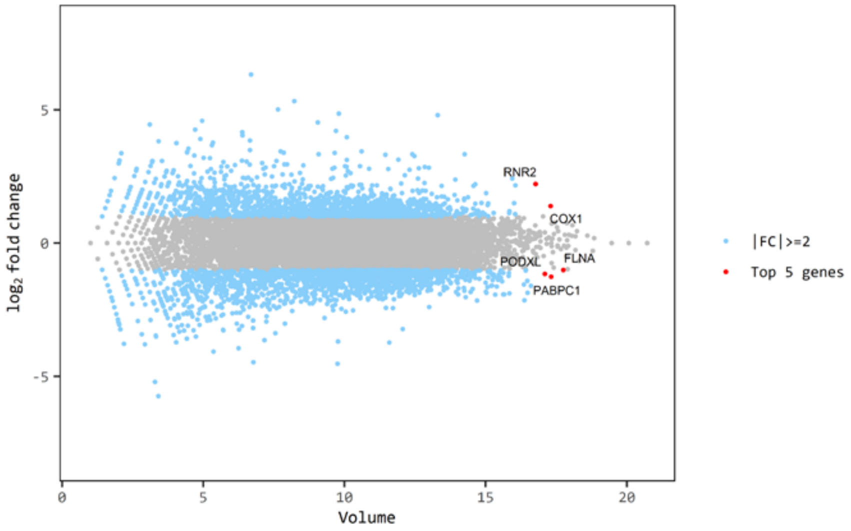

F - Volume Plot Between HB3_24h_vs_NC

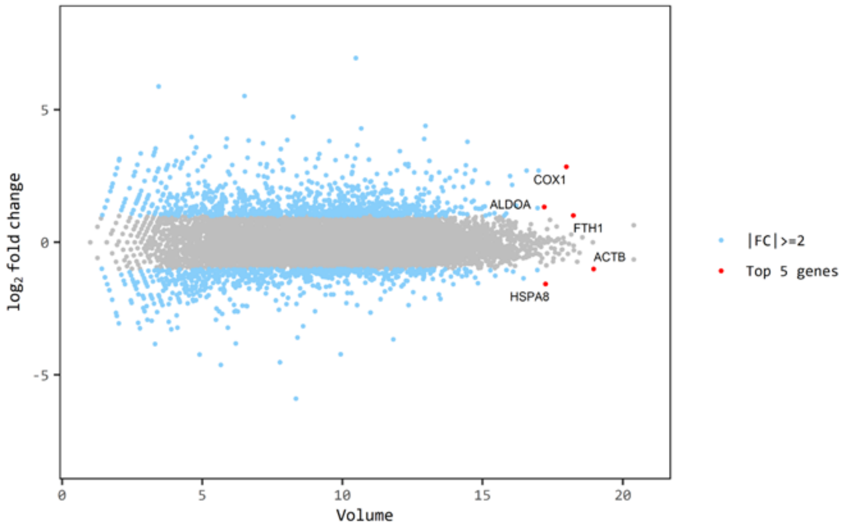

G - Volume Plot Between TNF-α_4h_vs_NC

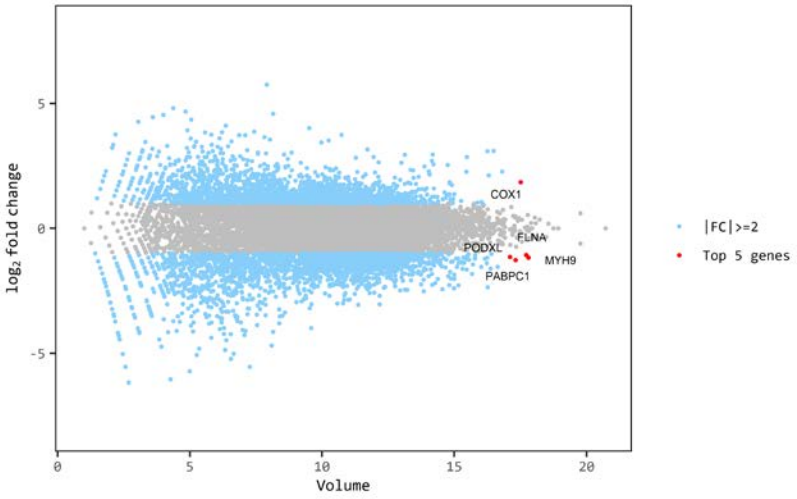

H - Volume Plot Between TNF-α _24h_vs_NC

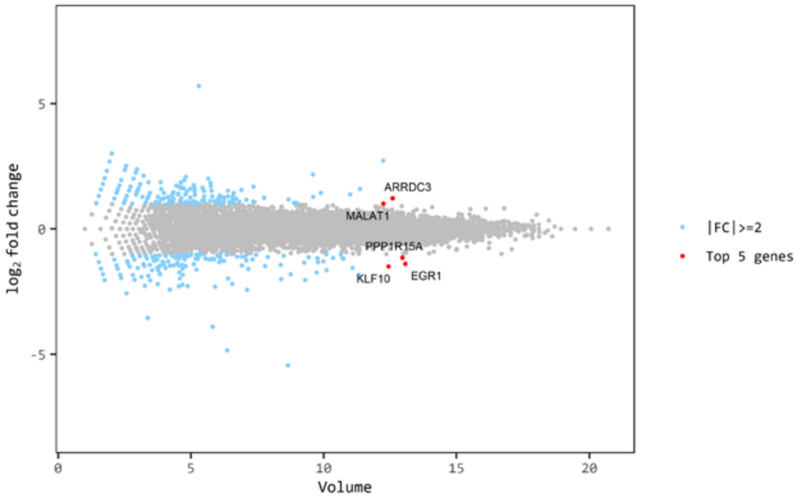

Figure S3: Volume plot. Expression volume is the geometric mean of two groups' expression levels. (volume = square-root (Control Normalized value * Test Normalized value)). To confirm the genes that show higher expression differences compared to the control according to expression volume, a volume plot is drawn. (X-axis: Volume, Y-axis: log2 Fold Change). For example, even though fold change might differ by 2-fold-change, the genes with higher expression volume may be more credible. Red dot ● means the top five genes by volume, which satisfy |fc| >= 2. The samples in the analysis are HBMEC without stimulation, used as negative control (NC), HBMEC with stimulation, working as a positive control, TNF at 4 h (TNF_4h) and 24 h (TNF_24h). HBMEC stimulated with Dd2 (Dd2_4h, Dd2_24h), HB3 (HB3_4h, Hb3_24h),

and 3D7 (3D7_4h, 3D7_24h) strains of *P. falciparum* parasites at a time point of 4 h and 24 h, respectively. The volume plot between NC and (A)3D7 4H, (B) 3D7 24H, (C) Dd2 4H, (D) Dd2 24H, (E) HB3 4H, (F) HB3 24H, (G) TNF 4H, (H) TNF 24H.

A

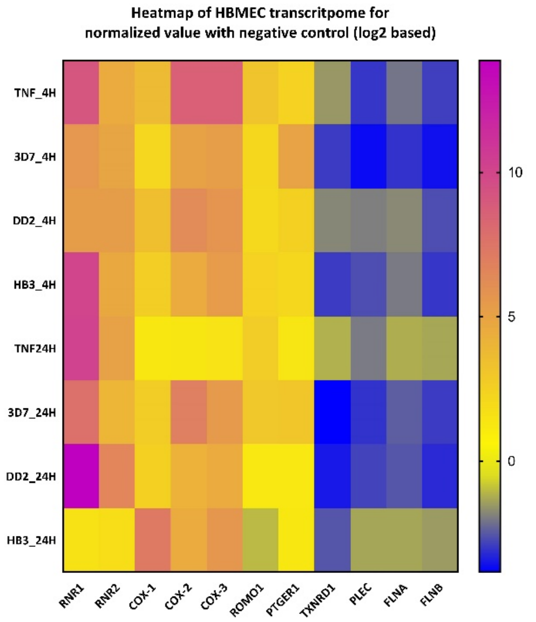

Heatmap of HBMEC transcritpome for
normalized value with negative control (log2 based)
TNF_4H
3D7_4H
DD2_4H
HB3_4H
TNF24H
3D7_24H
DD2_24H
HB3_24H
RNR1
RNR2
COX-1
COX-2
COX-3
ROMO1
PTGER1
TXNRD1
PLEC
FLNA
FLNB
10
5
0

B

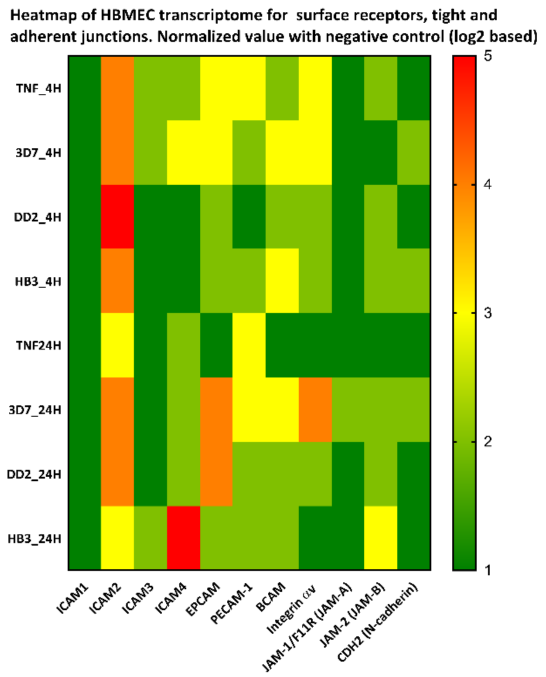

Heatmap of HBMEC transcriptome for surface receptors, tight and
adherent junctions. Normalized value with negative control (log2 based)
TNF_4H
3D7_4H
DD2_4H
HB3_4H
TNF24H
3D7_24H
DD2_24H
HB3_24H
ICAM1
ICAM2
ICAM3
ICAM4
EPCAM
PECAM-1
BCAM
Integrin αv
JAM-1/F11R (JAM-A)
JAM-2 (JAM-B)
CDH2 (N-cadherin)
5
4
3
2
1

C

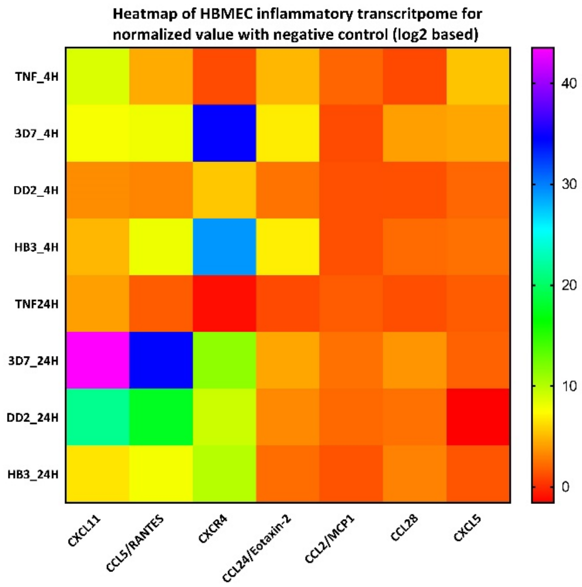
Heatmap of HBMEC inflammatory transcritpome for normalized value with negative control (log2 based)
TNF_4H
3D7_4H
DD2_4H
HB3_4H
TNF24H
3D7_24H
DD2_24H
HB3_24H
CXCL11
CCL5/RANTES
CXCR4
CCL24/Eotaxin-2
CCL2/MCP1
CCL28
CXCL5
40
30
20
10
0

D

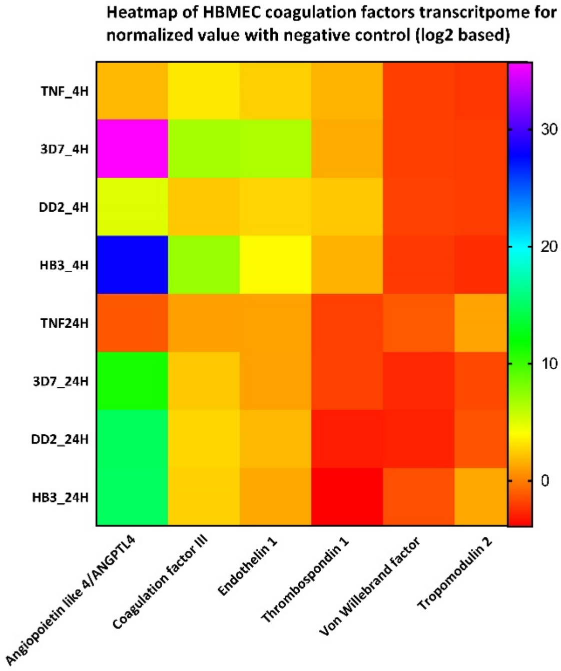
Heatmap of HBMEC coagulation factors transcritpome for normalized value with negative control (log2 based)
TNF_4H
3D7_4H
DD2_4H
HB3_4H
TNF24H
3D7_24H
DD2_24H
HB3_24H
Angiopoietin like 4/ANGPTL4
Coagulation factor III
Endothelin 1
Thrombospondin 1
Von Willebrand factor
Tropomodulin 2
30
20
10
0

E

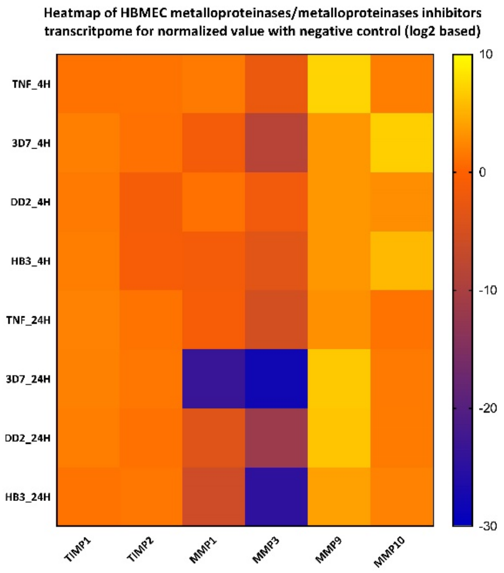

Figure S4: Heatmaps from HBMEC transcriptomic analysis. The heatmaps from HBMEC transcriptomic analysis using Dd2, HB3, 3D7 strains of *Plasmodium falciparum* parasites and TNF-α, at 4 h and 24 h, Dd2 4H, Dd2 24H, HB3 4H, HB3 24H, 3D7 4H, 3D7 24H, TNF_4h, and TNF_24h, respectively. **A)** Heatmap of Ribosomal 45S Cluster 1 (*RNR1*), Ribosomal 45S Cluster 2 (*RNR2*), Cyclooxygenase 1 (*COX-1*), *COX-2* and *COX-3*, Reactive Oxygen Species (*ROS*) Modulator 1 (*ROMO1*), Prostaglandin E Receptor 1 (*PTGER1*), Thioredoxin Reductase 1 (*TXNRD1*), Plectin (*PLEC*), Filamin A (*FLNA*), Filamin B (*FLNB*) are proteins coding genes. Genes that were downregulated are in blue, and up-regulated are in pink. **B)** Heatmap of transcriptomic expression of gene receptors I*CAM-1, ICAM-2, ICAM-3, ICAM-4, EPCAM, PECAM-1, BCAM, Integrin αV, F11R/JAM-A/JAM-1, JAM2/JAM-B, CDH2/N-cadherin* genes. Genes that were downregulated are in green and the ones up-regulated are in red. **C)** Heatmap of cytokines and chemokines. The data is presented in fold-change of *CXCL11, CCL5/RANTES, CXCR4, CCL24/Eotaxin-2, CCL2/MCP-1, CCL28,* and *CXCL5* genes. Genes that were downregulated are in blue and the ones up-regulated are in pink. **(D)** Heatmap of coagulation cascade genes. *ANGPTL4 genes* that encodes Angiopoietin-like 4 protein, *coagulation factor III*, *endothelin-1*, *thrombospondin-1*, Von Willebrand factor (*vWF*), and *Tmod2*) gene that encodes tropomodulin 2. **E)** Heatmap of metalloproteinases (MMP) and tissue

inhibitor metalloproteinase (TIMP). The data is presented in fold-change of *MMP-1, MMP-3, MMP-10, MMP-11, MMP-1, TIMP1,* and *TIMP-2* genes. The fold-change ratio of gene expression is based on negative control levels. Genes that were downregulated are in blue and the ones up-regulated are in yellow.

	PECAM-1	ICAM-1	CD36	EPCR	N- cadherin	JAM-A	IαVβ3
TNF-α 4H	1.104; ±0.034; $p<0.05$						

TNF-α 24H	1.189; ±0.063; *p<0.05*	1.349; ±0.141; *p<0.05*					
3D7 4H			1,302; ±0,098; *p<0.05*				0.889; ±0.032; *p<0.05*
3D7 24H			1.795; ±0.192; *p<0.0001*	1.461; ±0.142; *p<0.01*		1.153; ±0.051; *p<0.05*	1.373; ±0.125; *p<0.05*
Dd2 4H					(0.933; ±0.026; *p<0.05*		0.843; ±0.032; *p<0.001*
Dd2 24H			1.513; ±0,081; *p<0.01*			1.228; ±0.061; *p<0.001*	
HB3 4H							0,898; ±0,0371; *p<0.05*
HB3 24H			1.445; ±0.088; *p<0.01*			1.153; ±0.052; *p<0.0001*	

Table S1: Significant results from receptor expression of HBMEC. The results express the ratio of receptor expression in comparison to the negative control (NC) levels, the ± standard error of the mean and the statistical value (*p*).

	cxcl11	*RANTES*	*cxcr4*	*eotaxin-2*	*MCP-1/ccl2*	*ccl28*	*Cxcl5*	*angptl4*	*TSP-1*	*vWF*	*tropomodulin-2*	*MMP9*
TNF-α 4H	fc≥8	fc≥3	-	fc≥2	fc≥2	-	fc≥5	-	-	fc ⩽ -2	fc⩽ -2	fc≥6
TNF-α 24H	fc≥4	-	-	-	-	-	-	-	fc≤ -2	-	fc≤ -2	fc≥3
3D7 4H	fc≥7	fc≥3	fc≥5	fc≥6	fc≥4	fc≥4	fc≥4	fc⩾30	-	fc ⩽ -2	fc⩽ -2	fc≥3
3D7 24H	fc≥43	fc≥7	fc≥5	fc≥2	fc≥2	fc≥3.5	-	fc⩾10	-	fc ⩽ -2	-	fc≥6

Dd2 4H	fc≥3	fc≥3	fc≥5	fc≥2	-	fc≥2	fc≥2		fc≥2	fc ⩽ -2	fc⩽ -2	fc≥3
Dd2 24H	fc≥21	fc≥7	fc≥5	fc≥2	fc≥2	fc≥2	-	fc⩾10	fc ⩽ -3	fc ⩽ -2	-	fc≥3
HB3 4H	fc≥4	fc≥3	fc≥5	fc≥6	fc≥2	fc≥2	fc≥2	fc⩾20	-	fc ⩽ -2	fc⩽ -2	fc≥3
HB3 24H	fc≥6	fc≥7	fc≥5	fc≥2	fc≥3	fc≥3	-	fc⩾10	fc≤ -3	-	-	fc≥3

Table S2: **Significant fold-change from** HBMEC transcriptome. The fold-change ratio ofgene expression is based on negative control levels.

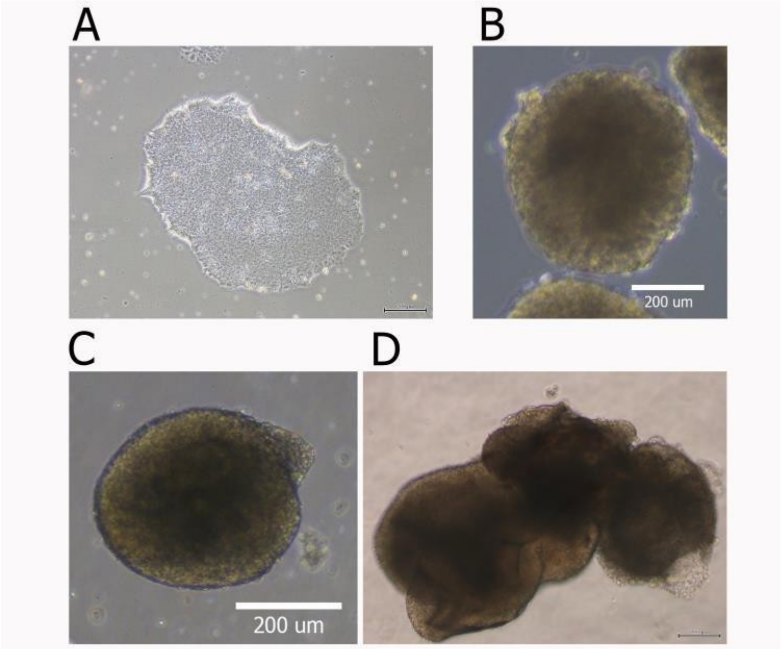

Figure S5: Bright field images from human brain organoids culture system. **A)** iPSCs culture used to generate brain organoids. **B)** Embryoid bodies (EB) formation after 6 days of spontaneous differentiation **C)** Neurospheres (NPCs) were formed after 11 days using a neural induction medium. **D)** Brain organoids with 30 days of post-differentiation. Scale bar: 200 μm.

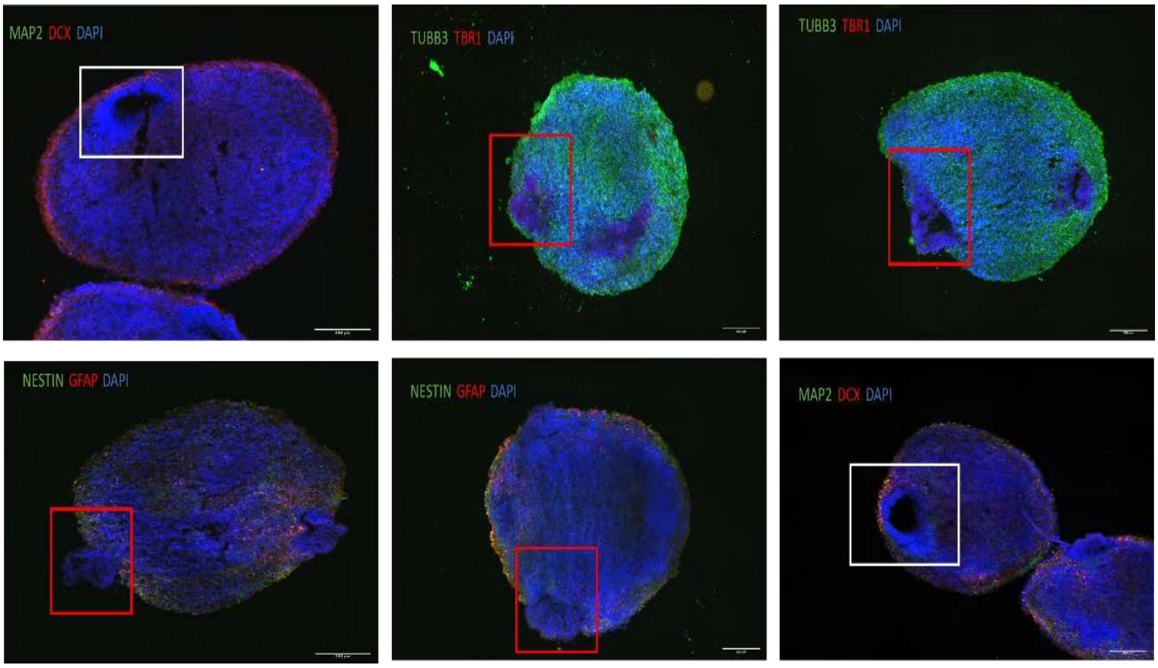

Figure S6: The neural tubes and heterogeneous regions of human brain organoids*in vitro***.** Representative immunostaining of human brain organoids with 45 days post-differentiation, in cryosections with 10-15 μm. Scale bars: 200 μm. Heterogeneous regions (red square) were similar to the choroid plexus architecture and neural tubes (white square). Nuclear staining with DAPI (Blue), neural stem cell marker (Nestin) (Green), the neuronal marker MAP2 (Green), human astrocytic marker, GFAP (Red), cortical-layer neurons TUBB3 (Green), newborn neurons marker DCX (Red) and pre-plate marker Tbr1 (Red). Brain organoids stimulated with secretome from HBMEC without any stimulation were called modified media (BO_MM), which means that HBMEC were in stimulated with half HBMEC culturemedia, and half *P. falciparum* parasites culture media. Brain organoids that grow only

with brain organoid media (BO), which works as a negative control, were also analyzed. The brain organoids were submitted to under several secretome conditions, such as with TFN-α stimulation, which works as a positive control (BO_TNF), three different wild-type strains, 3D7, Dd2, HB3 of *P. falciparum* parasites induction, at 4 h (BO_3D7_4H, BO_Dd2_4H, BO_HB3_4H) and 24 h (BO_3D7_24H, BO_Dd2_24H, BO_HB3_24H).

A

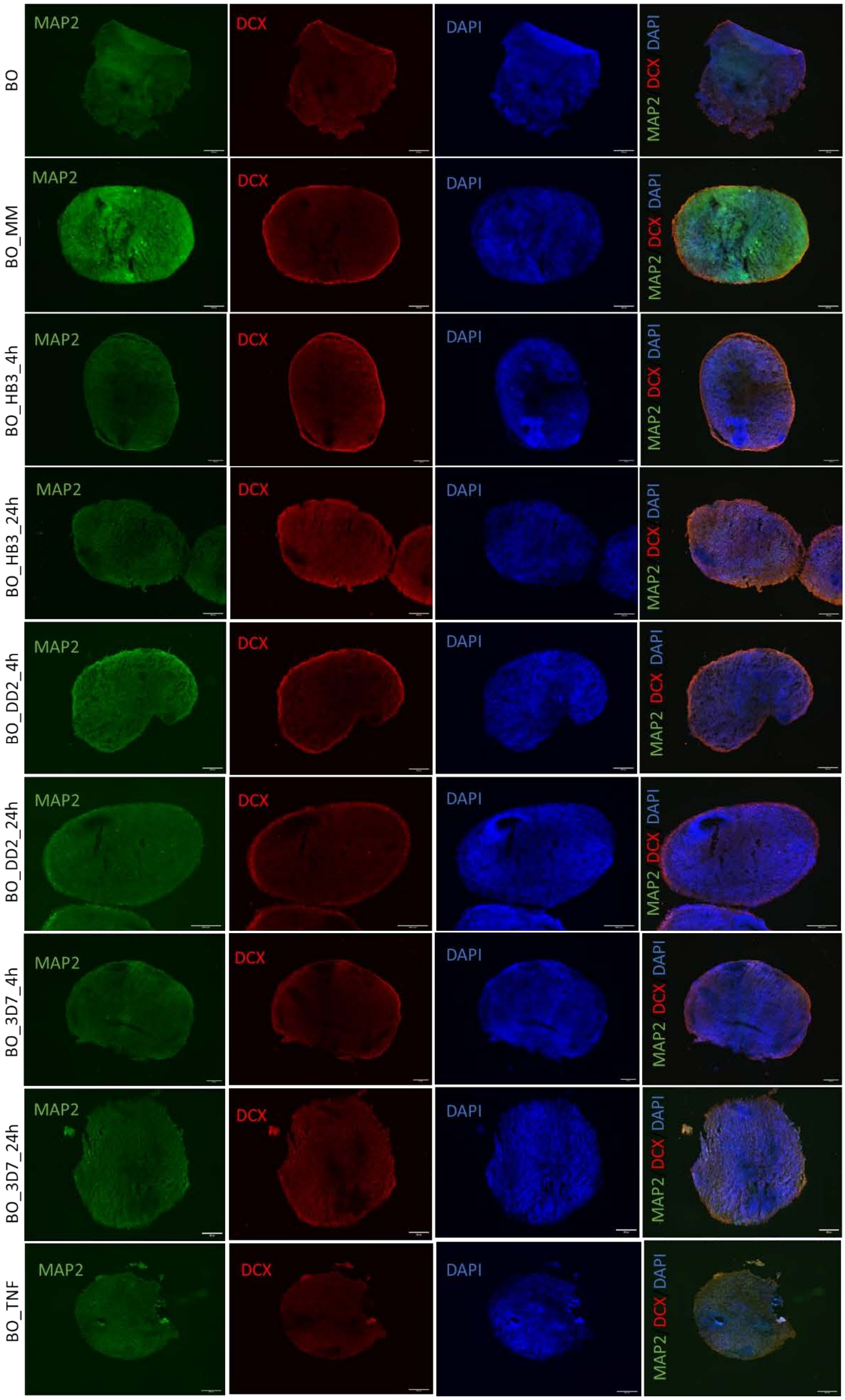

MAP2
DCX
DAPI
MAP2 DCX DAPI
BO
BO_MM
BO_HB3_4h
BO_HB3_24h
BO_DD2_4h
BO_DD2_24h
BO_3D7_4h
BO_3D7_24h
BO_TNF

B

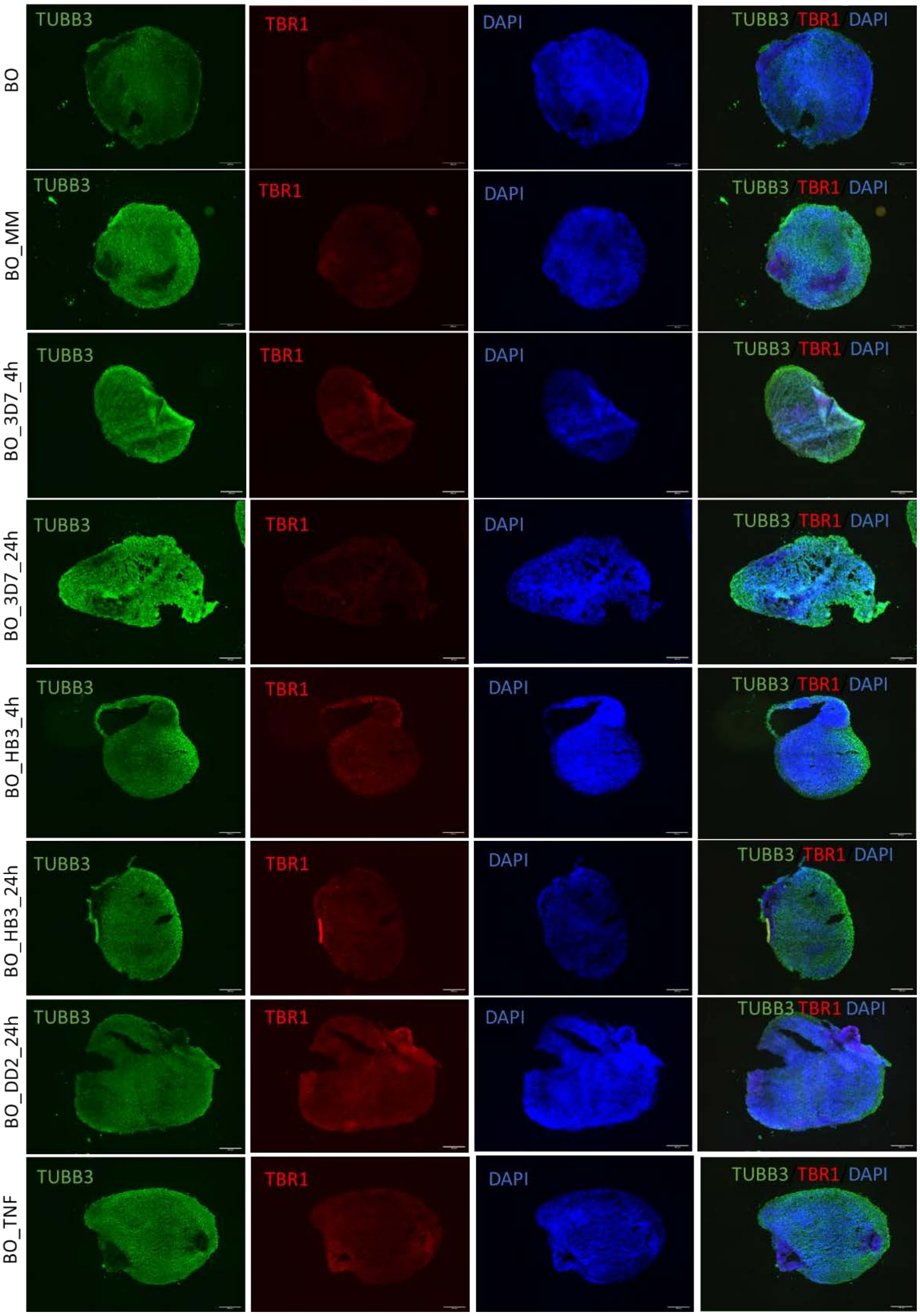
TUBB3
TBR1
DAPI
TUBB3 TBR1 DAPI
BO
BO_MM
BO_3D7_4h
BO_3D7_24h
BO_HB3_4h
BO_HB3_24h
BO_DD2_24h
BO_TNF

C

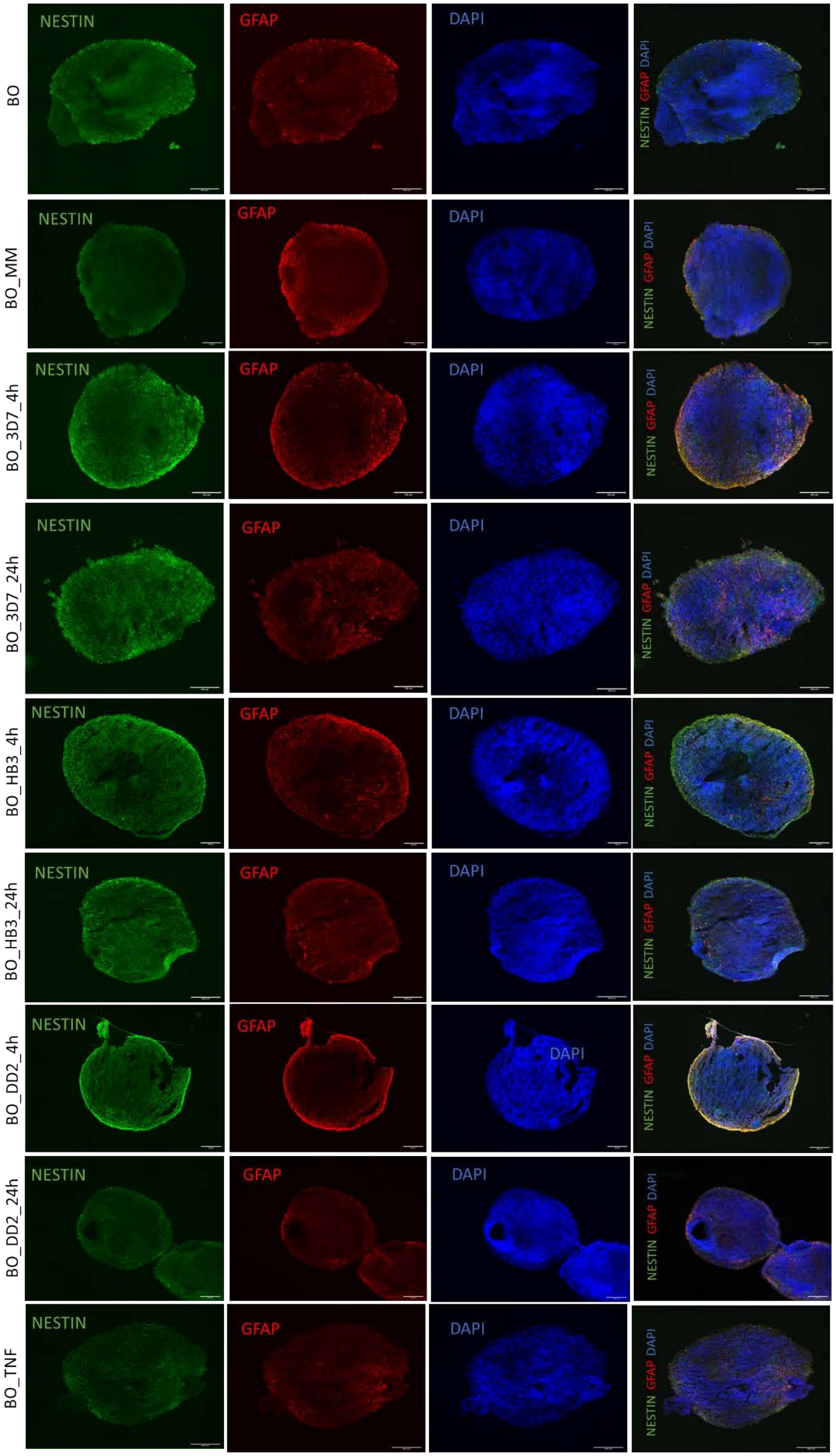
NESTIN
GFAP
DAPI
NESTIN GFAP DAPI
BO
BO_MM
BO_3D7_4h
BO_3D7_24h
BO_HB3_4h
BO_HB3_24h
BO_DD2_4h
BO_DD2_24h
BO_TNF

D

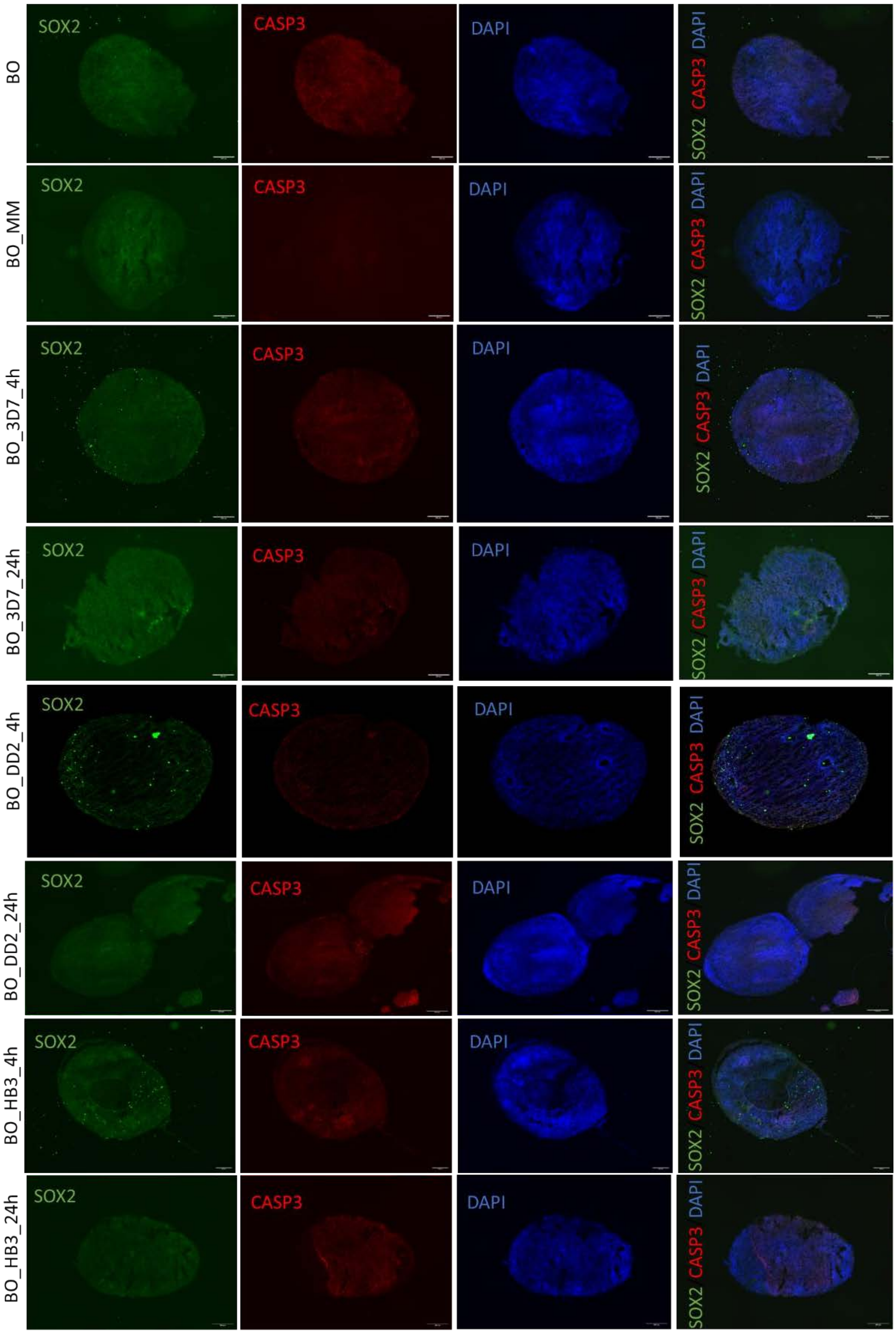

Figure S7: The expression of neuronal and glial proteins in human brain organoids. Representative immunostaining of human brain organoids with 45 days post-differentiation, in cryosections with 10-15 µm. Scale bars: 200 µm. Nuclear staining with DAPI (Blue). Brain organoids stimulated with secretome from HBMEC

were called modified media (BO_MM), which means that HBMEC were cultured with half HBMEC culture media, and half *P. falciparum* parasites culture media. Brain organoids that grow only with brain organoid media (BO) work as a negative control. The brain organoids were submitted to under several secretome conditions in the previous stimulation with HBMEC, such as with TFN-α stimulation, which works as a positive control (BO_TNF), three different wild-type strains, 3D7, Dd2, HB3 of *P. falciparum* parasites induction, at time points of 4h (BO_3D7_4H, BO_Dd2_4H, BO_Hb3_4H) and 24h (BO_3D7_24H, BO_Dd2_24H, BO_Hb3_24H), respectively. (A) Staining for the neuronal marker MAP2 (Green) revealing neuronal layer and for newborn neurons marker doublecortin (DCX) (Red). (B) Staining for cortical-layer neurons by tubulin beta 3 (TUBB3) (Green) marker, showing a sizeable cortical region. The pre-plate marker TBR1 (Red) for early-born neurons. (C) Immunostaining for a neural stem cell marker (Nestin) (Green) and glial fibrillary acidic protein (GFAP, human astrocytic marker) (Red). (D) Immunostaining with neural progenitor cells (NPC) marker SOX2 for the ventricular zone (VZ) and apoptosis marker Caspase 3 (CASP3). It showed the presence of SOX2+ cells and some apoptotic cell death (CASP3). It was possible to verify the presence of cavities in the brain organoids.

A

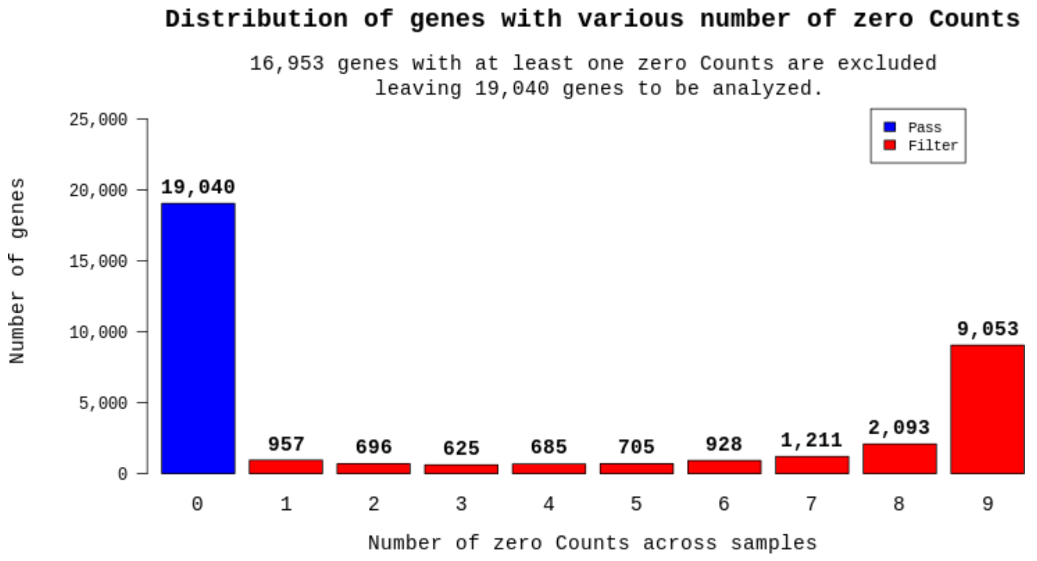

Distribution of genes with various number of zero Counts
16,953 genes with at least one zero Counts are excluded
leaving 19,040 genes to be analyzed.
Pass
Filter
Number of genes
25,000
20,000
15,000
10,000
5,000
0
19,040
957
696
625
685
705
928
1,211
2,093
9,053
0
1
2
3
4
5
6
7
8
9
Number of zero Counts across samples

B

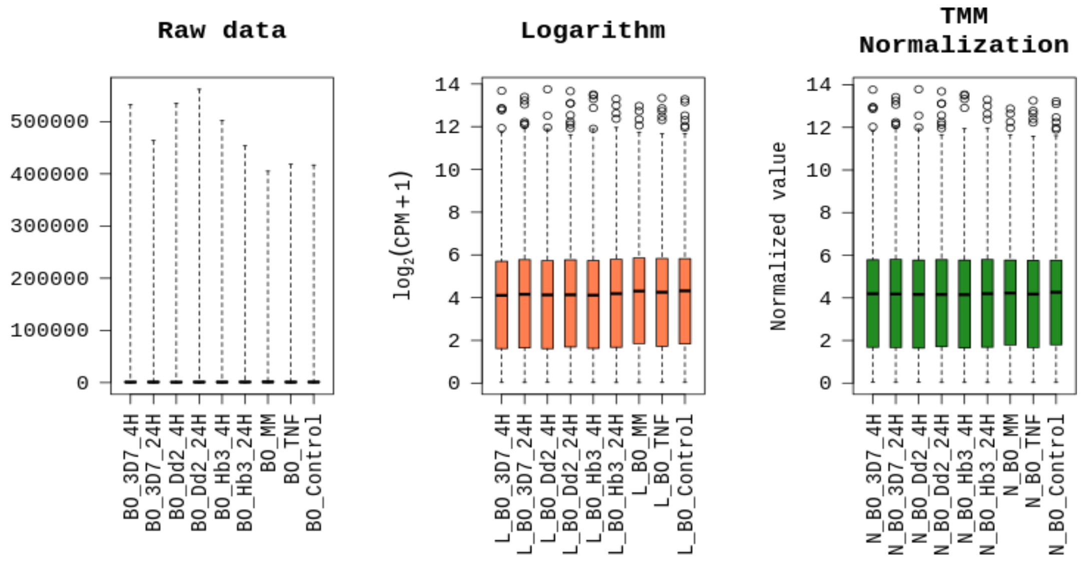

Raw data
Logarithm
TMM
Normalization
500000
400000
300000
200000
100000
0
14
12
10
8
6
4
2
0
log2(CPM+1)
Normalized value
BO_3D7_4H
BO_3D7_24H
BO_Dd2_4H
BO_Dd2_24H
BO_Hb3_4H
BO_Hb3_24H
BO_MM
BO_TNF
BO_Control
L_BO_3D7_4H
L_BO_3D7_24H
L_BO_Dd2_4H
L_BO_Dd2_24H
L_BO_Hb3_4H
L_BO_Hb3_24H
L_BO_MM
L_BO_TNF
L_BO_Control
N_BO_3D7_4H
N_BO_3D7_24H
N_BO_Dd2_4H
N_BO_Dd2_24H
N_BO_Hb3_4H
N_BO_Hb3_24H
N_BO_MM
N_BO_TNF
N_BO_Control

C

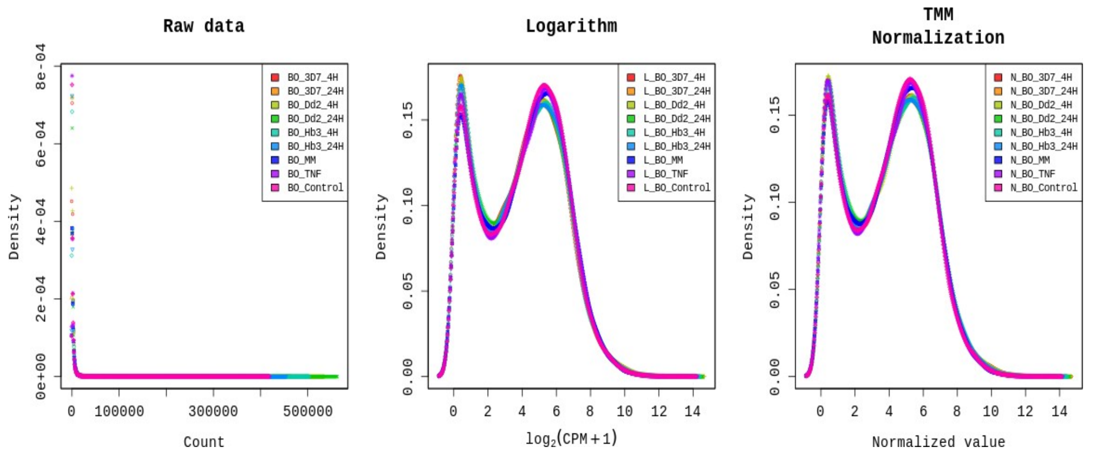

Raw data
Logarithm
TMM
Normalization
Density
8e-04
6e-04
4e-04
2e-04
0e+00
0
100000
300000
500000
Count
0.15
0.10
0.05
0.00
0
2
4
6
8
10
12
14
log2(CPM+1)
Normalized value
BO_3D7_4H
BO_3D7_24H
BO_Dd2_4H
BO_Dd2_24H
BO_Hb3_4H
BO_Hb3_24H
BO_MM
BO_TNF
BO_Control
L_BO_3D7_4H
L_BO_3D7_24H
L_BO_Dd2_4H
L_BO_Dd2_24H
L_BO_Hb3_4H
L_BO_Hb3_24H
L_BO_MM
L_BO_TNF
L_BO_Control
N_BO_3D7_4H
N_BO_3D7_24H
N_BO_Dd2_4H
N_BO_Dd2_24H
N_BO_Hb3_4H
N_BO_Hb3_24H
N_BO_MM
N_BO_TNF
N_BO_Control

D

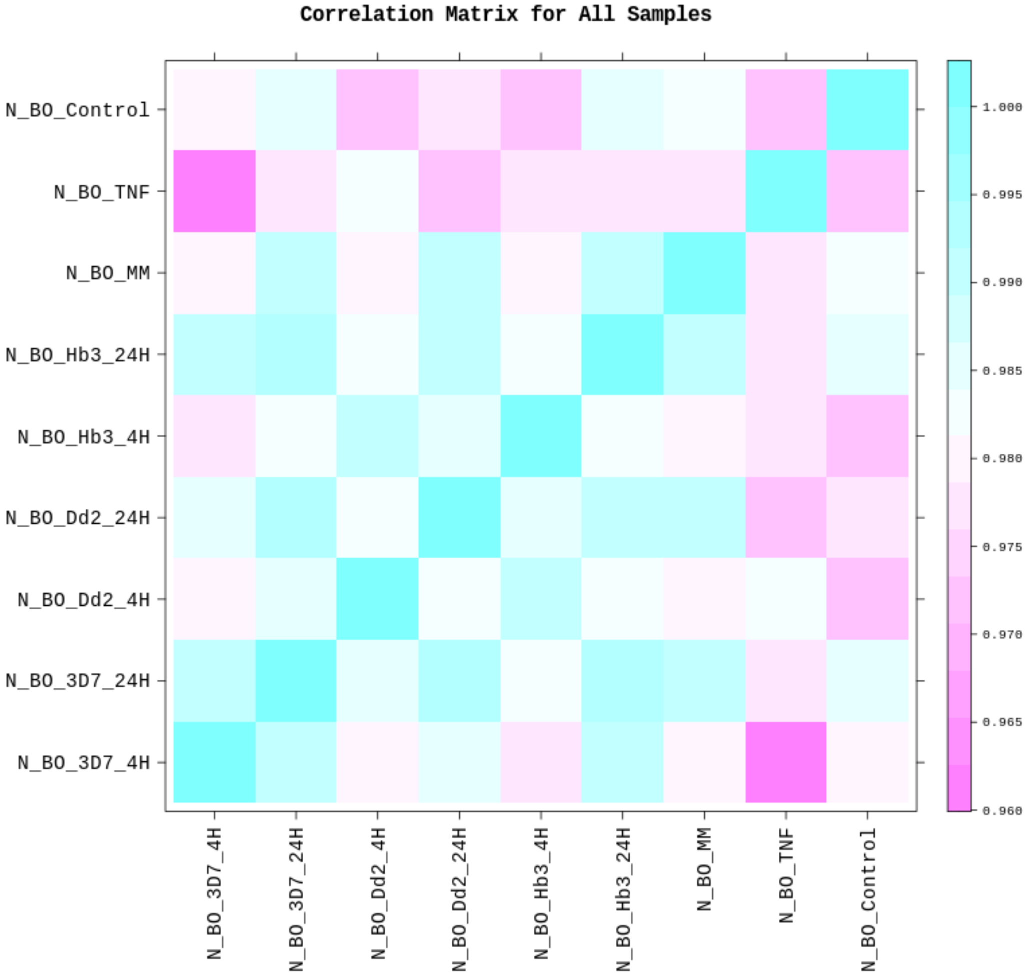

Figure S8: Brain organoids transcriptome data. To better understand the influence of secretome in brain organoids, from *P. falciparum* infection in the activation of HBMEC, single-cell RNA sequencing (scRNA-seq) of brain organoids was performed. The brain organoids transcriptomes analyzed were under several secretome conditions, such as with TFN-α stimulation, which works as a positive control (BO_TNF), three different wild-type strains, 3D7, Dd2, HB3 of *P. falciparum* parasites induction, at time points of 4h (BO_3D7_4H, BO_Dd2_4H, BO_HB3_4H) and 24h (BO_3D7_24H, BO_Dd2_24H, BO_HB3_24H), respectively. Brain organoids stimulation with secretome from HBMEC without any stimulation are called modified media (BO_MM), which means that HBMEC were in cultured with half HBMEC culture media and half *P. falciparum* parasites culture media. Brain organoids that grow only with brain organoid media (BO_control), which works as negative control, were also analyzed. (A) The data quality is evaluated through QC for each gene (histogram): For 9 samples, if more than one FPKM value was 0, it was

not included in the analysis. Therefore, from a total of 35,993 genes, 21,168 were excluded, and only 14,825 genes were used for statistical analysis. (B) Box plot: Below boxplots show the corresponding sample's expression distribution based on a percentile (median, 50 percentile, 75 percentile, maximum, and minimum) based on raw signal (FPKM), Log2 transformation, and Quantile Normalization. (C) Density plot: Below density plots show the corresponding sample's expression distribution before and after raw signal (FPKM+1), Log2 transformation, and Quantile Normalization. (D) Correlation matrix between Samples: The similarity between samples is obtained through Pearson's coefficient of the sample's normalized value. For range: $-1 \leqslant r \leqslant 1$, the closer the value is to 1, the more similar the samples are. The correlation matrix of all samples is as follows (X-axis as control and Y-axis as a test is the average normalized value of the group).

A

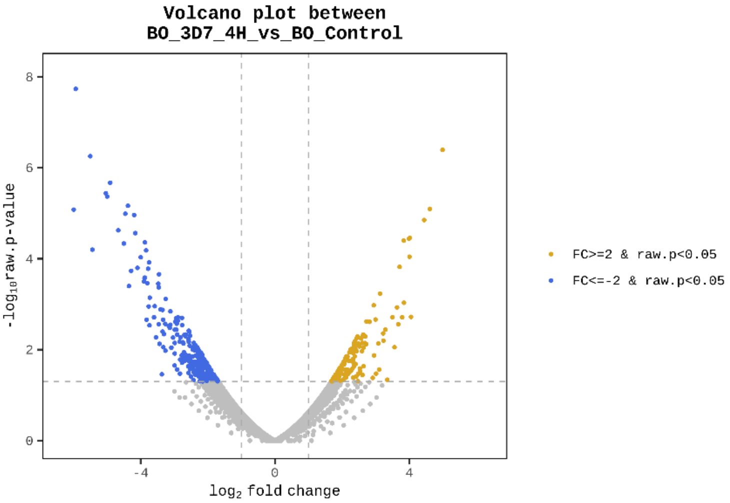

Volcano plot between
BO_3D7_4H_vs_BO_Control
-log10raw.p-value
log2 fold change
FC>=2 & raw.p<0.05
FC<=-2 & raw.p<0.05

B

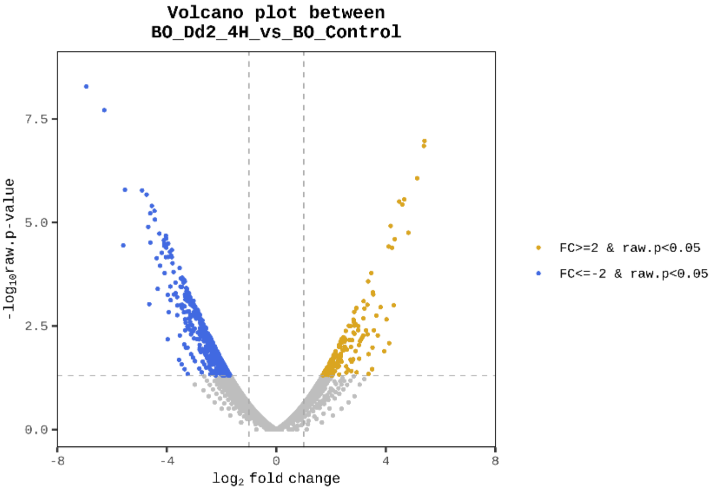

Volcano plot between
BO_Dd2_4H_vs_BO_Control
-log10raw.p-value
log2 fold change
FC>=2 & raw.p<0.05
FC<=-2 & raw.p<0.05

C

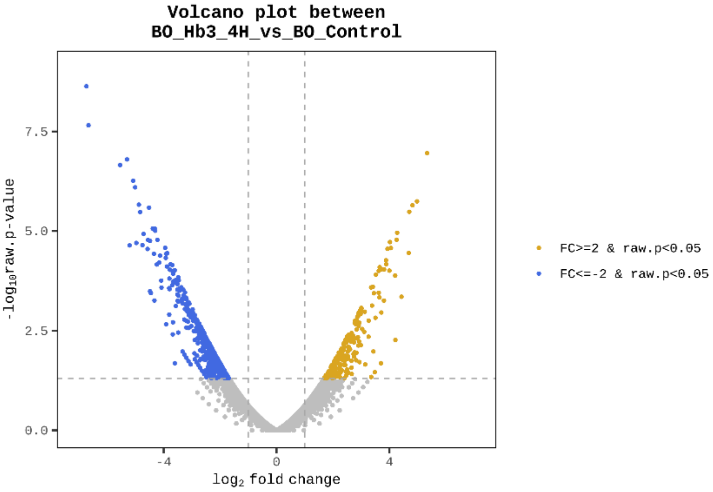

Volcano plot between
BO_Hb3_4H_vs_BO_Control
-log10raw.p-value
log2 fold change
FC>=2 & raw.p<0.05
FC<=-2 & raw.p<0.05

D

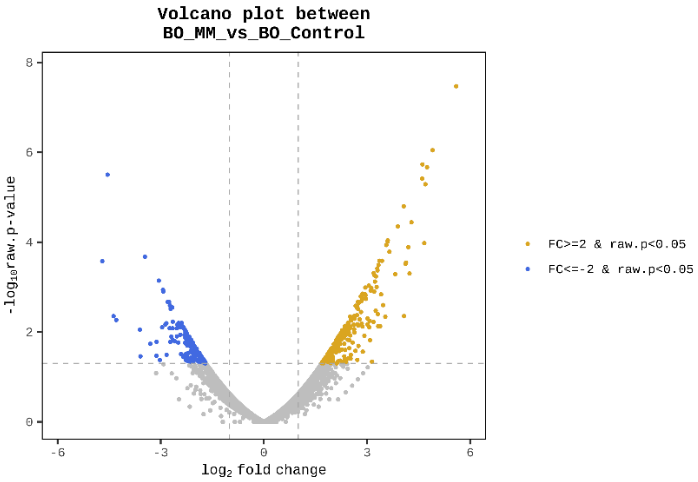
Volcano plot between
BO_MM_vs_BO_Control
-log10raw.p-value
log2 fold change
FC>=2 & raw.p<0.05
FC<=-2 & raw.p<0.05

E

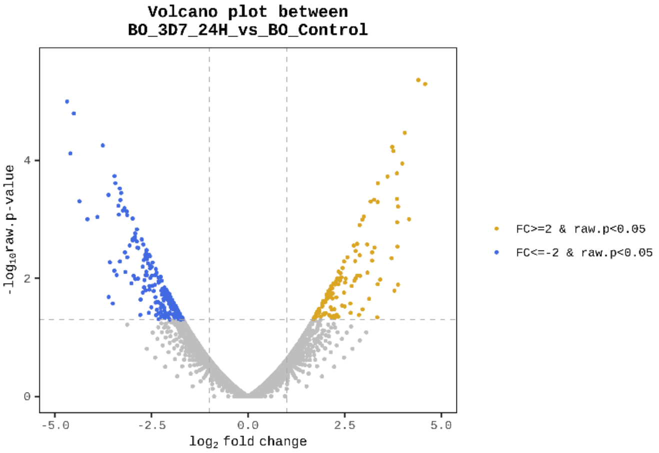
Volcano plot between
BO_3D7_24H_vs_BO_Control
-log10raw.p-value
log2 fold change
FC>=2 & raw.p<0.05
FC<=-2 & raw.p<0.05

F

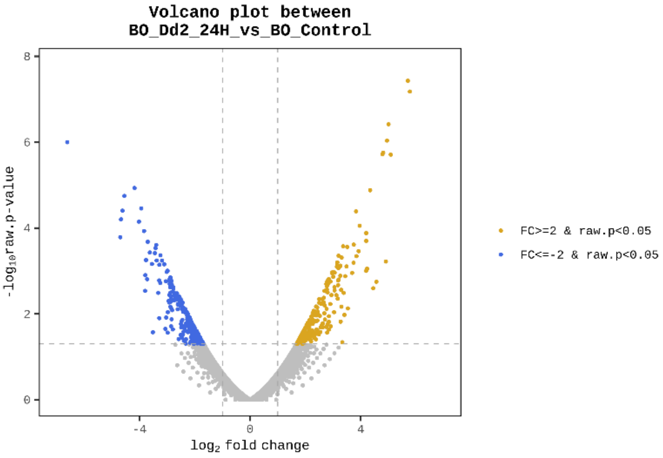
Volcano plot between
BO_Dd2_24H_vs_BO_Control
-log10raw.p-value
log2 fold change
FC>=2 & raw.p<0.05
FC<=-2 & raw.p<0.05

G

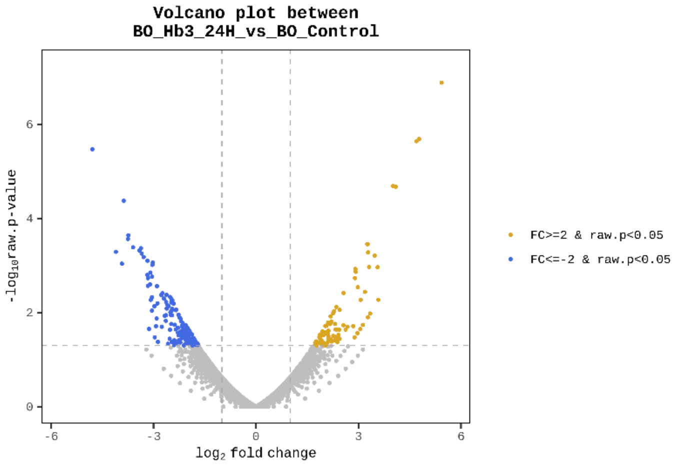

H

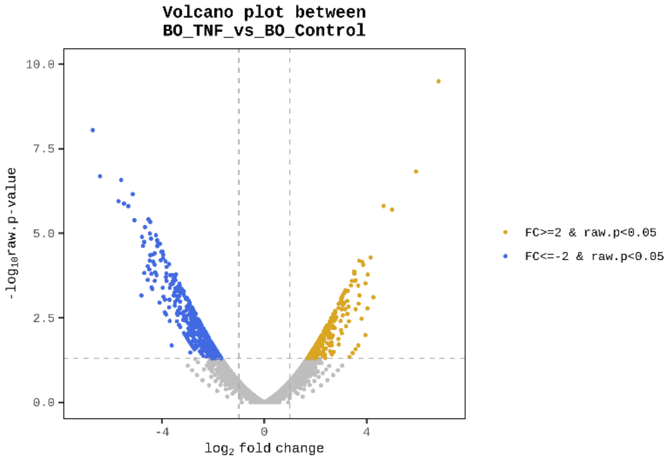

Figure S9: Volume plot. Expression volume is the geometric mean of two groups' expression levels. (volume = square-root (Control Normalized value * Test Normalized value)). To confirm the genes that show higher expression differences compared to the control according to expression volume, a volume plot is drawn. (X-axis: Volume, Y-axis: log2 Fold Change). For example, even though fold change might differ by 2-fold, the genes with higher expression volume may be more credible. ●: Top five genes by volume which satisfy |fc| >= 2. The brain organoids transcriptomes analyzed were under several secretome conditions, such as with TFN-α stimulation, which works as a positive control (BO_TNF), three different wild-type strains, 3D7, Dd2, HB3 of *P. falciparum* parasites induction, at time points of 4h (BO_3D7_4H, BO_Dd2_4H, BO_Hb3_4H) and 24h (BO_3D7_24H, BO_Dd2_24H,

BO_HB3_24H), respectively. Brain organoids stimulation with secretome from HBMEC without any stimulation are called modified media (BO_MM), which means that HBMEC were cultured with half HBMEC culture media and half *P. falciparum* parasites culture media. Brain organoids that grow only with brain organoid media (BO_control), which works as negative control, were also analyzed. The volume plot between BO and (A) 3D7 4H, (B) Dd2 4H, (C) HB3 4H, (D) MM, (E) 3D7 24H, (F) Dd2 24H, (G) HB3 24H, (H) TNF.

A

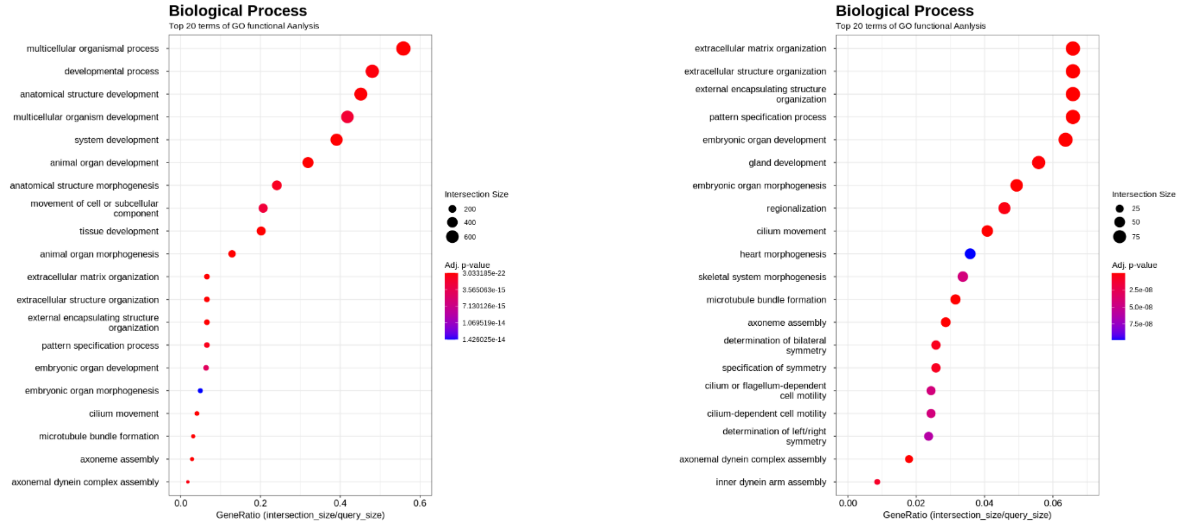

Biological Process
Top 20 terms of GO functional Aanlysis
multicellular organismal process
developmental process
anatomical structure development
multicellular organism development
system development
animal organ development
anatomical structure morphogenesis
movement of cell or subcellular component
tissue development
animal organ morphogenesis
extracellular matrix organization
extracellular structure organization
external encapsulating structure organization
pattern specification process
embryonic organ development
embryonic organ morphogenesis
cilium movement
microtubule bundle formation
axoneme assembly
axonemal dynein complex assembly
0.0 0.2 0.4 0.6
GeneRatio (intersection_size/query_size)
Intersection Size
200
400
600
Adj. p-value
3.033185e-22
3.565063e-15
7.130126e-15
1.069519e-14
1.426025e-14
Biological Process
Top 20 terms of GO functional Aanlysis
extracellular matrix organization
extracellular structure organization
external encapsulating structure organization
pattern specification process
embryonic organ development
gland development
embryonic organ morphogenesis
regionalization
cilium movement
heart morphogenesis
skeletal system morphogenesis
microtubule bundle formation
axoneme assembly
determination of bilateral symmetry
specification of symmetry
cilium or flagellum-dependent cell motility
cilium-dependent cell motility
determination of left/right symmetry
axonemal dynein complex assembly
inner dynein arm assembly
0.00 0.02 0.04 0.06
GeneRatio (intersection_size/query_size)
Intersection Size
25
50
75
Adj. p-value
2.5e-08
5.0e-08
7.5e-08

B

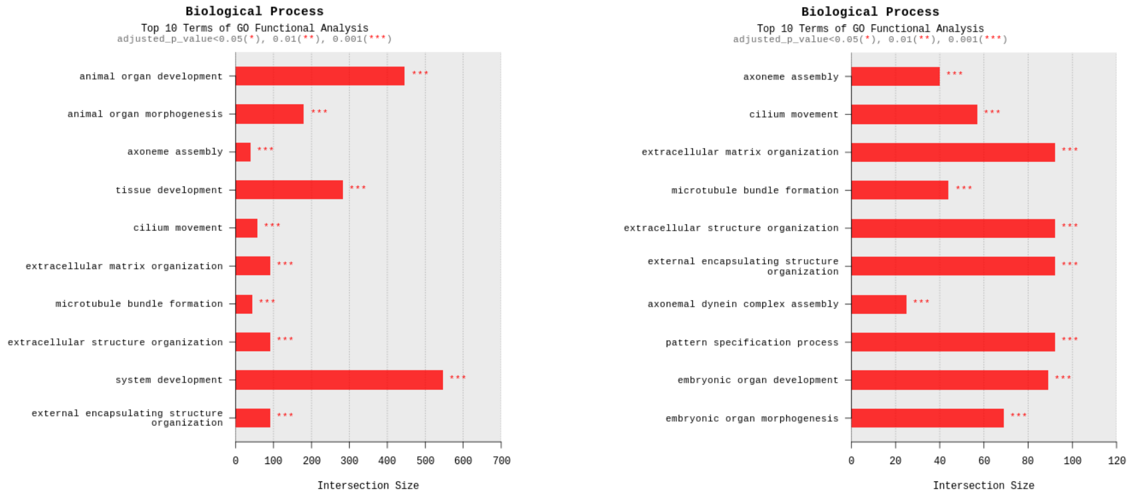

Biological Process
Top 10 Terms of GO Functional Analysis
adjusted_p_value<0.05(*), 0.01(**), 0.001(***)
animal organ development
animal organ morphogenesis
axoneme assembly
tissue development
cilium movement
extracellular matrix organization
microtubule bundle formation
extracellular structure organization
system development
external encapsulating structure organization
0 100 200 300 400 500 600 700
Intersection Size
Biological Process
Top 10 Terms of GO Functional Analysis
adjusted_p_value<0.05(*), 0.01(**), 0.001(***)
axoneme assembly
cilium movement
extracellular matrix organization
microtubule bundle formation
extracellular structure organization
external encapsulating structure organization
axonemal dynein complex assembly
pattern specification process
embryonic organ development
embryonic organ morphogenesis
0 20 40 60 80 100 120
Intersection Size

C

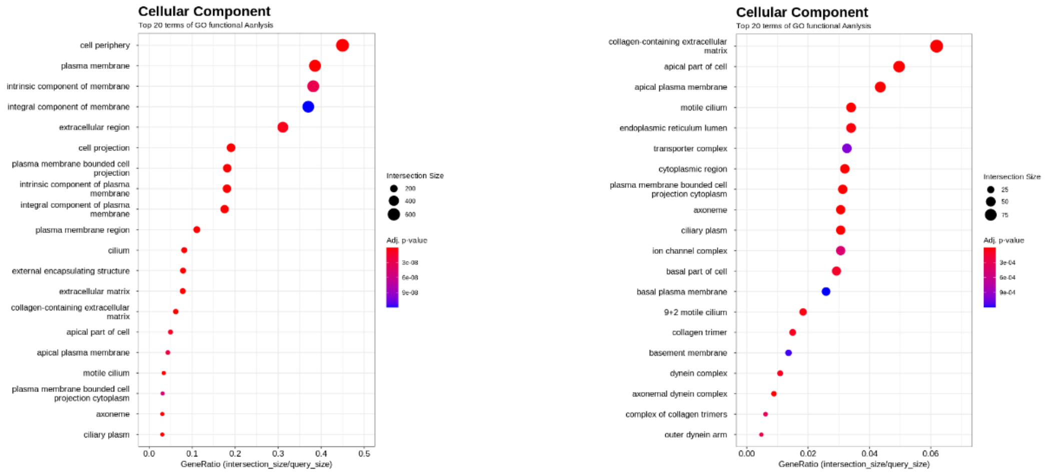

Cellular Component
Top 20 terms of GO functional Aanlysis
cell periphery
plasma membrane
intrinsic component of membrane
integral component of membrane
extracellular region
cell projection
plasma membrane bounded cell projection
intrinsic component of plasma membrane
integral component of plasma membrane
plasma membrane region
cilium
external encapsulating structure
extracellular matrix
collagen-containing extracellular matrix
apical part of cell
apical plasma membrane
motile cilium
plasma membrane bounded cell projection cytoplasm
axoneme
ciliary plasm
0.0 0.1 0.2 0.3 0.4 0.5
GeneRatio (intersection_size/query_size)
Intersection Size
200
400
600
Adj. p-value
3e-08
6e-08
9e-08
Cellular Component
Top 20 terms of GO functional Aanlysis
collagen-containing extracellular matrix
apical part of cell
apical plasma membrane
motile cilium
endoplasmic reticulum lumen
transporter complex
cytoplasmic region
plasma membrane bounded cell projection cytoplasm
axoneme
ciliary plasm
ion channel complex
basal part of cell
basal plasma membrane
9+2 motile cilium
collagen trimer
basement membrane
dynein complex
axonemal dynein complex
complex of collagen trimers
outer dynein arm
0.00 0.02 0.04 0.06
GeneRatio (intersection_size/query_size)
Intersection Size
25
50
75
Adj. p-value
3e-04
6e-04
9e-04

D

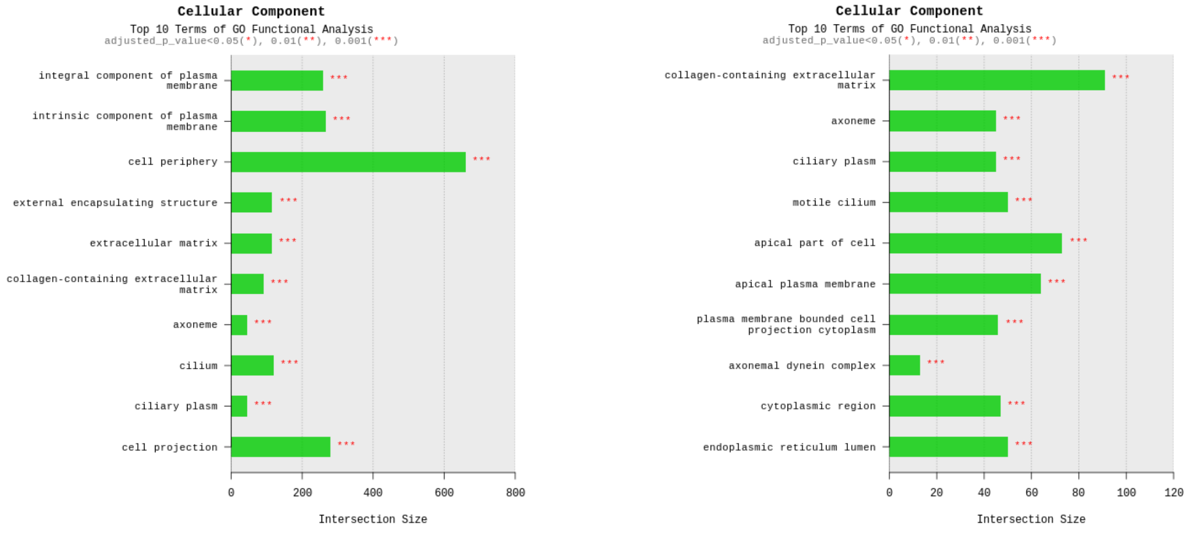

Cellular Component
Top 10 Terms of GO Functional Analysis
adjusted_p_value<0.05(*), 0.01(**), 0.001(***)
integral component of plasma membrane
intrinsic component of plasma membrane
cell periphery
external encapsulating structure
extracellular matrix
collagen-containing extracellular matrix
axoneme
cilium
ciliary plasm
cell projection
0
200
400
600
800
Intersection Size
Cellular Component
Top 10 Terms of GO Functional Analysis
adjusted_p_value<0.05(*), 0.01(**), 0.001(***)
collagen-containing extracellular matrix
axoneme
ciliary plasm
motile cilium
apical part of cell
apical plasma membrane
plasma membrane bounded cell projection cytoplasm
axonemal dynein complex
cytoplasmic region
endoplasmic reticulum lumen
0
20
40
60
80
100
120
Intersection Size

E

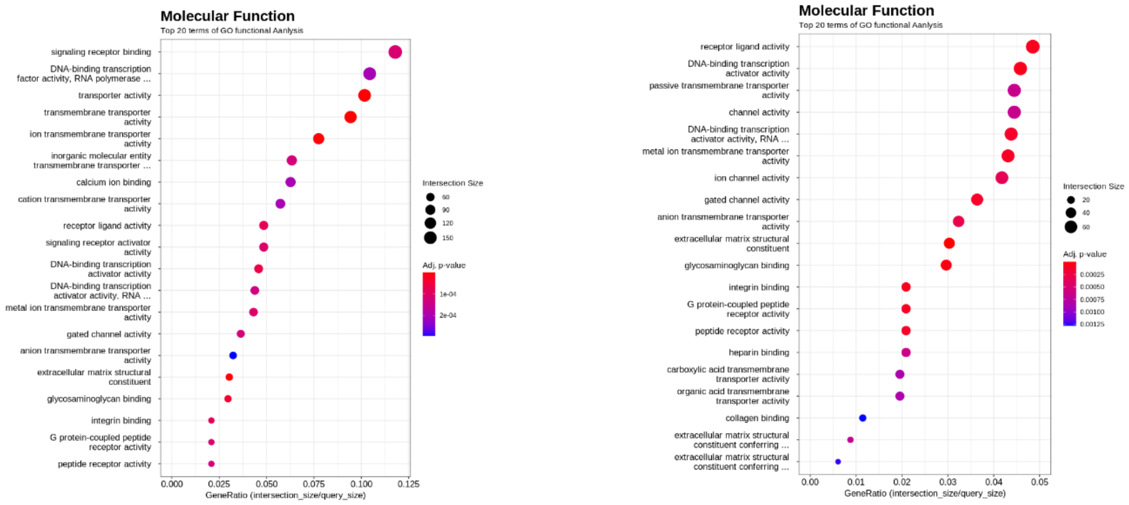

Molecular Function
Top 20 terms of GO functional Aanlysis
signaling receptor binding
DNA-binding transcription factor activity, RNA polymerase ...
transporter activity
transmembrane transporter activity
ion transmembrane transporter activity
inorganic molecular entity transmembrane transporter ...
calcium ion binding
cation transmembrane transporter activity
receptor ligand activity
signaling receptor activator activity
DNA-binding transcription activator activity
DNA-binding transcription activator activity, RNA ...
metal ion transmembrane transporter activity
gated channel activity
anion transmembrane transporter activity
extracellular matrix structural constituent
glycosaminoglycan binding
integrin binding
G protein-coupled peptide receptor activity
peptide receptor activity
Intersection Size
Adj. p-value
0.000
0.025
0.050
0.075
0.100
0.125
GeneRatio (intersection_size/query_size)
Molecular Function
Top 20 terms of GO functional Aanlysis
receptor ligand activity
DNA-binding transcription activator activity
passive transmembrane transporter activity
channel activity
DNA-binding transcription activator activity, RNA ...
metal ion transmembrane transporter activity
ion channel activity
gated channel activity
anion transmembrane transporter activity
extracellular matrix structural constituent
glycosaminoglycan binding
integrin binding
G protein-coupled peptide receptor activity
peptide receptor activity
heparin binding
carboxylic acid transmembrane transporter activity
organic acid transmembrane transporter activity
collagen binding
extracellular matrix structural constituent conferring ...
extracellular matrix structural constituent conferring ...
Intersection Size
Adj. p-value
0.00
0.01
0.02
0.03
0.04
0.05
GeneRatio (intersection_size/query_size)

F

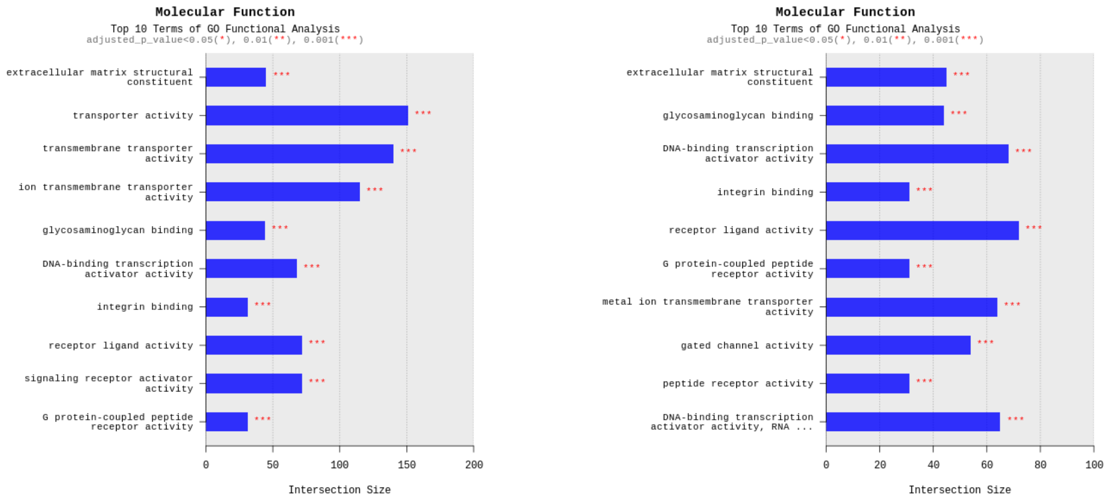

Molecular Function
Top 10 Terms of GO Functional Analysis
adjusted_p_value<0.05(*), 0.01(**), 0.001(***)
extracellular matrix structural constituent
transporter activity
transmembrane transporter activity
ion transmembrane transporter activity
glycosaminoglycan binding
DNA-binding transcription activator activity
integrin binding
receptor ligand activity
signaling receptor activator activity
G protein-coupled peptide receptor activity
0
50
100
150
200
Intersection Size
Molecular Function
Top 10 Terms of GO Functional Analysis
adjusted_p_value<0.05(*), 0.01(**), 0.001(***)
extracellular matrix structural constituent
glycosaminoglycan binding
DNA-binding transcription activator activity
integrin binding
receptor ligand activity
G protein-coupled peptide receptor activity
metal ion transmembrane transporter activity
gated channel activity
peptide receptor activity
DNA-binding transcription activator activity, RNA ...
0
20
40
60
80
100
Intersection Size

Figure S10: Transcriptome data about the biological process (BP), cellular component (CC), and molecular function (MF) of brain organoids within 45 daysof post-differentiation. DEG list was further analyzed with gProfiler for gene set enrichment analysis per biological process (BP), cellular component (CC), and molecular function (MF). The Gene Ratio is calculated as the ratio of intersection_size and query_size. The presented data is based on a fold-change of 2 and an adjusted *p-value.* (A) Top 20 terms of GO functional analysis of Biological Process. (B) Top 10 terms of GO functional analysis of Biological Process with a p-value of <0.05, 0.01, and 0.001. (C) Top 20 terms of GO functional analysis of Cellular component. (D) Top 10 terms of GO functional analysis of Biological Process with a *p-value* of <0.05, 0.01, and 0.001. (E) Top 20 terms of GO functional analysis of Molecular Function (F) Top 10 terms of GO functional analysis of Biological Process *with a p-value* of <0.05, 0.01, and 0.001.
